# Supplementary material for: Anticoagulation strategies in critical care for the treatment of atrial fibrillation: a protocol for a systematic review and meta-analysis
Source: BMJ Open. 2020 Oct 20;10(10):e037591. doi: 10.1136/bmjopen-2020-037591 (PMC7577029; doi:10.1136/bmjopen-2020-037591)
Supplement: Supplementary data [file bmjopen-2020-037591supp001.pdf]

Supplementary File: Search Strategy used to identify papers included in this review using the Healthcase Databases Advanced Search engine.

DATABASE	MESH TERMS	KEYWORDS (TI AND AB)
MEDLINE	ANTICOAGULANTS/ OR "4HYDROXYCOUMARINS"/ OR ABCIXIMAB/ OR ACENOCOUMAROL/ OR ANCROD/ OR ANTITHROMBINS/ OR BECAPLERMIN/ OR "BETA 2-GLYCOPROTEIN I"/ OR "BLOOD COAGULATION FACTOR INHIBITORS"/ OR "CITRIC ACID"/ OR DALTEPARIN/ OR "DERMATAN SULFATE"/ OR DEXTRANS/ OR DICUMAROL/ OR "EDETIC ACID"/ OR ENOXAPARIN/ OR "ETHYL BISCOUMACETATE"/ OR "FIBRIN FIBRINOGEN DEGRADATION PRODUCTS"/ OR GABEXATE/ OR HEPARIN/ OR "HEPARIN, LOWMOLECULAR-WEIGHT"/ OR HEPARINOIDS/ OR NADROPARIN/ OR "PENTOSAN SULFURIC POLYESTER"/ OR PHENINDIONE/ OR PHENPROCOUMON/ OR "PROTEIN C"/ OR "PROTEIN S"/ OR "SODIUM CITRATE"/ OR TINZAPARIN/ OR WARFARIN/ exp "BLOOD COAGULATION"/	(anticoagul* OR anti-coagul* OR warfarin OR heparin* OR antiplatelet* OR anti-platelet* OR "Direct Oral Anticoagul*" OR "DOAC*" OR "new oral anticoagul*" OR "NOAC*" OR apixaban OR dabigatran OR "dabigatran etexilate" OR rivaroxaban OR "vitamin K antagonist*" OR "VKA").ti,ab
	exp "ATRIAL FIBRILLATION"/	("atrial fibrillation" OR "AF" OR "atrial flutter*" OR "supraventricular tachy*" OR "SVT").ti,ab
	exp "CRITICAL CARE"/	("critical care" OR "Intensive care" OR "ITU" OR "critical* ill*" OR "high depend*" OR "intensive therapy").ti,ab
EMBASE	exp "ANTICOAGULANT AGENT"/	(anticoagul* OR anti-coagul* OR warfarin OR heparin* OR antiplatelet* OR anti-platelet* OR "Direct Oral Anticoagul*" OR "DOAC*" OR "new oral anticoagul*" OR "NOAC*" OR apixaban OR dabigatran OR "dabigatran etexilate" OR rivaroxaban OR "vitamin K antagonist*" OR "VKA").ti,ab
	exp "ATRIAL FIBRILLATION"/	("atrial fibrillation" OR "AF" OR "atrial flutter*" OR "supraventricular tachy*" OR "SVT").ti,ab
	"INTENSIVE CARE"/	("critical care" OR "Intensive care" OR "ITU" OR "critical* ill*" OR "high depend*" OR "intensive therapy").ti,ab
PUBMED		(anticoagul* OR anti-coagul* OR warfarin OR heparin* OR antiplatelet* OR anti-platelet* OR "Direct Oral Anticoagul*" OR "DOAC*" OR "new oral anticoagul*" OR "NOAC*" OR apixaban OR dabigatran OR "dabigatran etexilate" OR rivaroxaban OR "vitamin K antagonist*" OR "VKA").ti,ab
		("atrial fibrillation" OR "AF" OR "atrial flutter*" OR "supraventricular tachy*" OR "SVT").ti,ab
		("critical care" OR "Intensive care" OR "ITU" OR "critical* ill*" OR "high depend*" OR "intensive therapy").ti,ab

COCHRANE		(anticoag*)
		“atrial fibrillation” OR AF
		“Critical care” OR “Intensive care” OR “ITU” OR “high depend*” OR “HDU”
