# Supplementary material for: Anticoagulation strategies in critical care for the treatment of atrial fibrillation: a protocol for a systematic review and meta-analysis
Source: BMJ Open. 2020 Oct 20;10(10):e037591. doi: 10.1136/bmjopen-2020-037591 (PMC7577029; doi:10.1136/bmjopen-2020-037591)
Supplement: Author's manuscript [file bmjopen-2020-037591.draft_revisions.pdf]

BMJ Open is committed to open peer review. As part of this commitment we make the peer review history of every article we publish publicly available.

When an article is published we post the peer reviewers' comments and the authors' responses online. We also post the versions of the paper that were used during peer review. These are the versions that the peer review comments apply to.

The versions of the paper that follow are the versions that were submitted during the peer review process. They are not the versions of record or the final published versions. They should not be cited or distributed as the published version of this manuscript.

BMJ Open is an open access journal and the full, final, typeset and author-corrected version of record of the manuscript is available on our site with no access controls, subscription charges or pay-per-view fees (http://bmjopen.bmj.com).

If you have any questions on BMJ Open's open peer review process please email info.bmjopen@bmj.com

BMJ Open

Anticoagulation strategies in critical care for the treatment of atrial fibrillation: a protocol for a systematic review and meta-analysis

Journal:	*BMJ Open*
Manuscript ID	bmjopen-2020-037591
Article Type:	Protocol
Date Submitted by the Author:	10-Feb-2020
Complete List of Authors:	Johnston, Brian; University of Liverpool Faculty of Health and Life Sciences,<br>Nelson, Alexandra; University of Liverpool Faculty of Health and Life Sciences<br>Waite, Alicia; University of Liverpool Institute of Ageing and Chronic Disease<br>Lemma, Gedeon; University of Liverpool Faculty of Health and Life Sciences<br>Welters, Ingeborg; University of Liverpool Institute of Ageing and Chronic Disease,
Keywords:	INTENSIVE & CRITICAL CARE, Adult intensive & critical care < ANAESTHETICS, CARDIOLOGY, Anticoagulation < HAEMATOLOGY

SCHOLARONE™
Manuscripts

I, the Submitting Author has the right to grant and does grant on behalf of all authors of the Work (as defined in the below author licence), an exclusive licence and/or a non-exclusive licence for contributions from authors who are: i) UK Crown employees; ii) where BMJ has agreed a CC-BY licence shall apply, and/or iii) in accordance with the terms applicable for US Federal Government officers or employees acting as part of their official duties; on a worldwide, perpetual, irrevocable, royalty-free basis to BMJ Publishing Group Ltd ("BMJ") its licensees and where the relevant Journal is co-owned by BMJ to the co-owners of the Journal, to publish the Work in this journal and any other BMJ products and to exploit all rights, as set out in our licence.

The Submitting Author accepts and understands that any supply made under these terms is made by BMJ to the Submitting Author unless you are acting as an employee on behalf of your employer or a postgraduate student of an affiliated institution which is paying any applicable article publishing charge ("APC") for Open Access articles. Where the Submitting Author wishes to make the Work available on an Open Access basis (and intends to pay the relevant APC), the terms of reuse of such Open Access shall be governed by a Creative Commons licence – details of these licences and which Creative Commons licence will apply to this Work are set out in our licence referred to above.

Other than as permitted in any relevant BMJ Author's Self Archiving Policies, I confirm this Work has not been accepted for publication elsewhere, is not being considered for publication elsewhere and does not duplicate material already published. I confirm all authors consent to publication of this Work and authorise the granting of this licence.

For peer review only

Anticoagulation strategies in critical care for the treatment of atrial fibrillation: a protocol for a systematic review and meta-analysis

Authors

1) Brian W Johnston[1,2], 2) Alexandra Nelson[2], 3) Alicia C Waite[1,2], 4) Gedeon Lemma[2], 5) Ingeborg D Welters[1,2]

Corresponding author: Brian W Johnston, c/o Intensive Care Unit, Royal Liverpool and Broadgreen University Hospital, Prescott Street, L78XP, Contact Telephone: 0151 706 2410

Author Affiliations

1. University of Liverpool and The Royal Liverpool University Hospital, Liverpool Healthcare Partners, Liverpool, UK.
2. The University of Liverpool, Liverpool, UK.

Author contact details

1. Dr Brian. W Johnston, brian.johnston@liverpool.ac.uk, ORCID: 0000-0003-1634-3297
2. Miss Alexandra Nelson, hlanels2@liverpool.ac.uk
3. Dr Alicia C Waite, Alicia.Waite@liverpool.ac.uk, ORCID: 0000-0001-8734-994X
4. Mr Gedeon Lemma, hlglemma@liverpool.ac.uk
5. Prof Ingeborg D Welters, Ingeborg.welters@liverpool.ac.uk, ORCID: 0000-0002-3408-8798

Keywords

Atrial Fibrillation, Critical Illness, Intensive Care Unit, Anticoagulation,

Prospero Trials Registration Number: CRD42020158237

For peer review only

ABSTRACT

Introduction

Atrial fibrillation is the most common cardiac arrhythmia in critically ill patients and is associated with an increased risk of thromboembolic events and mortality. Oral anticoagulation for thromboembolism prophylaxis is a key component of managing atrial fibrillation in the general population and is recommended by NICE guidelines. However, assessment tools used to aid decision making about anticoagulation have not yet been validated in the critical care setting. There is a paucity of data assessing the impact of anticoagulation strategies on clinical outcomes in critically ill patients with atrial fibrillation. We present a protocol for a systematic review and meta-analysis to evaluate the effectiveness of anticoagulation strategies for atrial fibrillation used specifically in critical care.

Methods

We will conduct a systematic review of the literature by searching MEDLINE, EMBASE, CENTRAL and PubMed databases for articles published from inception until 2019. Studies reporting anticoagulation strategies for AF in adults (>18 years) admitted to a general critical care setting will be assessed for inclusion. Outcomes of interest will include, 1) percentage of patients commenced on anticoagulation in critical care for atrial fibrillation, 2) incidence of thromboembolism, 3) incidence of bleeding events, 4) ICU mortality, 5) hospital mortality, 6) ICU length of stay and 7) hospital length of stay. If sufficient data allows, we will conduct a meta-analysis of trials. Risk of bias will be assessed using the Cochrane Risk of Bias tool for randomised trials or the Newcastle-Ottowa Risk of Bias assessment tool for non-randomised studies. This protocol and subsequent systematic review will be reported following the Preferred Reporting Items for Systematic Reviews and Meta-Analyses checklist.

Ethics and dissemination

This proposed systematic review will include data extracted from published studies, therefore ethical approval is not required. The results of this review will be published in clinical specialty journals and presented at international meetings and conferences.

For peer review only - http://bmjopen.bmj.com/site/about/guidelines.xhtml

Prospero Trial Registration Number: CRD42020158237

Strengths and limitations of this study

- Atrial fibrillation is common in critical care and causes a significant disease burden, however, no anticoagulation treatment guidelines exist specifically for critically ill patients.
- Existing anticoagulation strategies have not been validated in the critically ill patients.
- A consensus on effective anticoagulation strategies to prevent thromboembolic events during critical illness has not been reached.
- This protocol describes a systematic review and meta-analysis of the existing literature regarding anticoagulation strategies for patients with atrial fibrillation in critical care.
- The results of this systematic review and meta-analysis will support further research and guide treatment decisions.

INTRODUCTION

Background

Atrial fibrillation (AF) is the most common cardiac arrhythmia in the critical care population. AF is characterised by erratic conduction of electrical impulses and uncoordinated contraction of the atria which ultimately increases the risk of heart failure, stroke and death.[1,2] Nearly one third of patients admitted to critical care have a diagnosis of pre-existing atrial fibrillation (PEAF) or develop new-onset atrial fibrillation (NOAF) during their admission.[2] AF can trigger rapid ventricular rates resulting in haemodynamic compromise and a loss of cardiac output. This may contribute to acute decompensation of already unstable critically ill patients and eventually lead to the increased morbidity and mortality associated with AF in critical care. [2,3]

In addition to the traditional risk factors for the development of AF, including advanced age, hypertension, ischaemic heart disease, heart failure and valvular disease; there are factors related to being critically ill that

For peer review only - http://bmjopen.bmj.com/site/about/guidelines.xhtml

predispose patients to the development of NOAF.[1,2] These factors include electrolyte abnormalities, hypoxaemia, adrenergic overstimulation, progressive autonomic dysfunction, acute systemic inflammation, sepsis and shock.[1,2] Changes in autonomic activity resulting from vasopressor administration as well as electrolyte disturbances are frequent among critically ill patients and can lead to increased atrial ectopic impulses and subsequent NOAF.[3] There is also evidence that central venous catheters induce mechanical irritation of the atria and may be a contributing factor in the development of NOAF.[1] The high incidence of AF in critical care is likely due to a combination of these traditional and critical illness related risk factors.[3]

Both PEAF and NOAF are associated with a myriad of complications in critical care. As a result of inefficient atrial systole, the reduced blood flow velocity in the left atria predisposes patients with AF to cardiac and systemic emboli.[4] Embolic events, such as ischaemic stroke, are common and disabling complications of AF and cause a significant disease burden.[5] AF in the critical care setting is associated with a twofold increased risk of stroke and a two to fivefold increased risk of mortality.[2,6]

Oral anticoagulation for thromboembolism prophylaxis is a key component of managing AF in the general population.[7] However, the risk-benefit tools used to aid decision making about anticoagulation, such as the CHA_2DS_2-VASc and HASBLED scores, have not been validated in critical care populations.[8,9] Decisions around anticoagulation strategies in critical care are complex and challenging. Critically unwell patients are at a significantly increased risk of bleeding, but may also of hypercoagulable, due to the abnormal haemostasis that is associated with critical illness.[9] Factors contributing to the acquired coagulopathy in critical care include severe sepsis, thrombocytopenia, haemodilution of clotting factors following blood transfusion, disseminated intravascular coagulation (DIC), acute kidney injury (AKI) and liver failure.[10] The potential need for urgent procedures or insertion of invasive devices, such as arterial lines and central venous catheters, poses an additional challenge in effectively anticoagulating these patients.[11] Combined with the added effect of anticoagulation itself, the high risk of bleeding may preclude safe therapeutic anticoagulation for AF in critical care patients.

Why is it important to undertake this review?

For peer review only

There is a paucity of data assessing the impact of anticoagulation strategies on clinical outcomes in critically unwell patients with AF.[12] A consensus on an effective anticoagulation strategy for thromboembolism prophylaxis has not yet been reached and current recommendations regarding anticoagulation therapy are largely based on observational studies and expert opinion.[3] We therefore have designed a protocol for a systematic review and meta-analysis of the literature around the anticoagulation for AF in the critically unwell and critical care setting. We will assess the existing literature to define anticoagulation strategies in critical care for both NOAF and PEAF. We anticipate that the results of this review will highlight areas were evidence is lacking, trigger further research and contribute to the development of new guidelines specific to the management of anticoagulation in patients with AF in the critical care setting.

Objective

To conduct a systematic review and meta-analysis of the literature on the effectiveness of anticoagulation strategies for AF in critical care and identify the associated risks and benefits of therapeutic anticoagulation for AF.

METHODS

This systematic review will be conducted in accordance with The Cochrane Collaboration principles of Systematic Reviews and will be reported following the Preferred Reporting Items for Systematic Reviews and Meta-Analyses (PRISMA) guidelines. [13,14]

This protocol has been registered with the International Prospective Register of Systematic Reviews (PROSPERO) database. PROSPERO registration number: CRD42020158237

Inclusion criteria

Type of studies

All quantitative studies that report anticoagulation strategies for AF, in an adult critical care setting will be assessed for inclusion. Eligible studies will include randomised, non-randomised, prospective and retrospective studies.

For peer review only - http://bmjopen.bmj.com/site/about/guidelines.xhtml

Eligible studies must include both a cohort of patients with AF who were anticoagulated and a cohort of patients with AF who were not anticoagulated. All studies must be conducted in a critical care setting that is not service specific, for example general medical, general surgical or mixed intensive care units (ICU). Studies based on high dependency units (HDU) will be included also.

Phenomenon of interest

Studies must include patients who have been in AF for >48 hours, and may include both PEAF and NOAF. Studies must describe outcomes associated with the chosen anticoagulation strategy including the outcomes of interest described below.

Population

Studies that include adult patients admitted to the ICU types specified above will be assessed for inclusion. For the purpose of this review, an adult is defined as ≥18 years.

Type of intervention and comparator(s)

Interventions:

Eligible studies will include any of the following treatments:

1) Warfarin
2) Any other vitamin K antagonist
3) Unfractionated heparin (UFH)
4) Treatment dose low molecular weight heparin (LMWH)
5) Factor Xa inhibitors (e.g Edoxaban, Apixaban, Rivaroxaban)
6) Direct thrombin (Factor IIa) inhibitors (Dabigatran, Inogatran, Melagatran, Argatroban)
7) Any combination of the above interventions

Comparators:

For peer review only

Comparators will include:

1) Any of the interventions above
2) Placebo
3) Standard care where it does not include anticoagulation

Exclusion criteria

Types of studies

Qualitative studies, case studies, editorials, letters, abstract only reports, reviews and commentaries that do not include original information will be excluded from this review.

Studies of cohorts that have undergone or plan to undergo cardiothoracic surgery, permanent pacemaker insertion or surgical ablation will be excluded. Studies based on service-specific ICUs, for example cardiac, cardiothoracic surgical or neurosurgical units, will be excluded in addition to studies based on acute medical units or in emergency departments.

Phenomenon of interest

Studies will be excluded if patients have been commenced on any anticoagulation therapy for a reason other than AF and cannot be disaggregated from the entire cohort. Studies that include patients with an inherited or pre-existing bleeding disorder or clotting disorder will also be excluded.

Population

Studies of participants under 18 years or pregnant women will be excluded.

Outcome measures

Primary outcome measure:

1) Percentage of patients commenced on anticoagulation for AF (NOAF and/or PEAF) in a critical care setting including but not limited to warfarin, LMWH, UFH, Factor Xa inhibitors, Factor IIa inhibitors.

For peer review only

Secondary outcome measures:

1) Incidence of thromboembolic events (defined as stroke, mesenteric ischaemia, acute limb ischaemia, pulmonary embolism) during critical care admission
2) Development of a major haemorrhage event, defined as per the study authors
3) ICU length of stay (LOS)
4) ICU mortality
5) 28-day mortality
6) 90-day mortality
7) 365-day mortality
8) Use of risk stratification scores for anticoagulation decision making (such as CHA_2DS_2-VASc and HASBLED)
9) Severity scores of critical illness (e. g. APACHE II and SOFA)

Search strategy

We will engage the services of a health information specialist, and a comprehensive broad literature search will be conducted. Medical subject headings will be used to identify papers that matched the search index and relevant selected free text terms will also be used.

The search strategies identified will focus on the population (critically ill), the participants (patients with AF) and the intervention (any anticoagulation strategy mentioned previously). The search strategy will not be limited by outcomes studied in order to broaden the scope of eligible papers.

Database searches

We will search the following databases:

1) MEDLINE Ovid (from inception until 2019), Medline Ovid Epub Ahead of Print, and Medline Ovid In-Process

For peer review only

2) EMBASE Ovid (from inception until 2019)

3) The Cochrane Central Register of Controlled Trials

4) PUBMED

5) ClinicalTrials.gov

MEDLINE, EMBASE and PUBMED will be access via NICE Healthcare Database Advanced Search (HDAS) using OpenAthens. We will screen referenced papers following full text screening and any review articles identified during the screening process. We will screen for relevant conference proceedings and include any potentially relevant studies. A full description of the search strategy that will be used in HDAS (supplementary file). We will conduct a further search of the literature using the same search strategy to identify any studies that are published between commencing title and abstract screening and data extraction.

Citation management and screening

Results from searches in all databases will be exported to Endnote X9 (Clarivate analytics) and duplicates will be removed. All citations will be imported into the Covidence systematic review platform (Veritas Health Innovation, Melbourne, Australia). Titles and abstracts will be screened in duplicate by two independent reviewers for relevance to the review and eliminated as appropriate. Full text articles that are not excluded after title and abstract screening, will be screened against the inclusion and exclusion criteria by two independent reviewers. Any discrepancies or conflicts in the screening process will be resolved by discussion and subsequent input of a third reviewer. Primary reason for exclusion will be documented in Covidence and the screening process will be documented in a PRISMA flowchart (Figure 1).

Data extraction and management

Data will be extracted in duplicate by two independent reviewers using a standardised, pre-piloted data extraction form.

For peer review only

Information, including the following characteristics, will be extracted from studies:

1) Study design and methodology, including title, authors, journal, publication date, study type, study period and number of participants
2) All inclusion criteria previously mentioned
3) Population characteristics, including age, sex, ICU admission diagnosis, location, co-morbidities, illness severity scores
4) Recruitment procedures
5) Interventions
6) The primary and secondary outcome measures and reported findings of the study

Any discrepancies within data extraction will be resolved through discussion and consultation with a third reviewer. We will contact authors if clarification regarding the data or methodology is required. If data cannot be obtained from the authors, the impact will be discussed as a limitation of the review.

Risk of bias assessment

Risk of bias will be assessed using a modified Newcastle-Ottawa Scale (mNOS), a scoring system for non-randomised trials.[15] mNOS uses a system by which the study is judged on three broad perspectives: the selection of the study groups, the comparability and the ascertainment of outcome of interest.

Included randomised controlled trials will be assessed with the Cochrane Collaborations Risk of Bias (RoB) tool using the criteria outlined in the Cochrane Handbook of Systematic Reviews of Interventions.[16] The RoB tool assesses the following domains: selection, comparability, outcome performance and will be presented in table format.

Data Synthesis and Analysis

A description of included studies will be reported in evidence tables and discussed in the text. We will report participant characteristics, interventions, clinical outcomes and methodological quality.

For peer review only

Primary outcomes will be reported as the percentage of patients that received anticoagulation for any AF in critical care. If sufficient data allows, we will report the percentage of patients commenced on anticoagulation according to type of AF (NOAF and/or PEAF).

We will present dichotomous data as number of participants experiencing the outcome with OR and 95% confidence intervals. For continuous outcome data, we will extract arithmetic means and standard deviations with 95% confidence intervals for each outcome, together with the numbers analysed in each group. We will also extract medians and ranges where provided.

If possible, data from individual studies will be pooled and presented visually by Forest plot. We will assess for heterogeneity between studies using the chi-squared test and report the degree of heterogeneity using the I^2 statistic in RevMan (Review Manager, 2014). We will undertake a meta-analysis of included studies if the assumption of homogeneity is met and it is appropriate to do so, and we will perform a random effects analysis to account for differences in effect sizes between studies. Meta-analysis will be conducted in the Cochrane Collaborations RevMan (Review Manager, 2014) software. If the characteristics of included studies are heterogeneous, we will describe the findings via a narrative synthesis.

We will assess the certainty of our evidence for each outcome according to the Grading of Recommendations Assessment, Development and Evaluation (GRADE) approach.[17] GRADE methodology assesses the certainty in evidence across the domains of risk of bias, consistency, directness, precision and publication bias. The certainty of evidence will be reported as high, moderate, low or very low.

Subgroup analysis

If we extract sufficient data, we will undertake subgroup analysis, assessing the following:

1. Type of anticoagulant therapy
2. NOAF versus PEAF
3. Sepsis versus non-sepsis
4. Illness severity scores (APACHE II score, SOFA score)
5. Risk scores for bleeding and thromboembolic events (HASBLED, CHA_2DS_2-VASc)

For peer review only

6. Medical versus surgical ICU admission diagnosis

Discussion

This systematic review will assess the evidence available for anticoagulation strategies used in the management of AF in critically ill patients. AF affects up to 25% of critical care admissions, hence the need for systematic evaluation of anticoagulation strategies used in critical care and their impact on patient outcomes. [18] AF is associated with an increased risk of thromboembolic complications, but scoring systems used to aid decision making on whether and how-to anticoagulate patients with AF have not been validated for use in the critical care setting. This review will enable a comparison of the anticoagulation strategies used for both PEAF and NOAF in critical care and aims to assess outcomes including thromboembolic events, haemorrhagic events, length of stay (LOS) in critical care and mortality on ICU.

Based on an initial scoping review of the literature, we believe that this is the first systematic review to consider anticoagulation strategies used in the treatment of AF in the critically ill patient cohort. We aim for this review to provide an evidence base upon which recommendations can be made, to ultimately reduce the impact of AF on patient outcomes during and after critical care admission. We will publish the results of this review in clinical specialty journals and we present it at international meetings and conferences. We will also make our report available via our web- based repository and update our PROSPERO entry with the web address details.

FOOTNOTES

Author's contribution

The protocol was conceived and designed by IW, BWJ and AW. AN will conduct primary screening and data collection, reviewed by BWJ and GL. Data extraction, analysis and preparation of the manuscript will be conducted by AN and BWJ. IW, BWJ, AW, GL and AN will read and approve the final manuscript.

Funding

For peer review only

This research received no specific grant from any funding agency in the public, commercial or not-for-profit sectors.

Conflicts of interest

None declared.

Data Sharing Statement

All data is included in the manuscript.

Provenance and peer review

Not commissioned; externally peer reviewed.

Patient consent

Not required.

Patient and public involvement

There was no patient or public involvement in the planning or writing of this protocol

Figure Legend

Figure 1: PRISMA flowchart of studies selected in the systematic review

For peer review only

REFERENCES

1. Malik A, Candilio L, Hausenloy DJ. Atrial fibrillation in the intensive care setting. *Journal of the Intensive Care Society.* 2013;14(2):141-9.

2. Bosch NA, Cimini J, Walkey AJ. Atrial Fibrillation in the ICU. *Chest*. 2018;154(6):1424-34.

3. Walkey AJ, Hogarth DK, Lip GYH. Optimizing Atrial fibrillation management from ICU and beyond. *Chest*. 2015;148(4):859-64.

4. Nadzeya Kuzniatsova, Lip GYH. Prevention of Thromboembolism in Atrial Fibrillation Patients. *European Cardiology Review*. 2011;7(1):37-43.

5. Gorczyca I, Wozakowska-Kaplon B, Starzyk K et al. Evaluation of the recommended prevention of thrombosis in hospitalised patients with atrial fibrillation and high thromboembolism risk. *Kardiol Pol.* 2018;76(3):625-32.

6. Arrigo M, Bettex D, Rudiger A. Management of atrial fibrillation in critically ill patients. *Crit Care Res Pract*. 2014;2014:840615-.

7. Gilligan DM, Ellenbogen K, Epstein E. Atrial Fibrillation: Management 2014 [updated August 2014.] Available from: https://www.nice.org.uk/guidance/cg180

8. Kanji S, Williamson DR, Yaghchi BM et al. Epidemiology and management of atrial fibrillation in medical and noncardiac surgical adult intensive care unit patients. *J Crit Care.* 2012;27(3):326.e1-8.

9. Poli D, Antonucci E, Pengo V et al. Comparison of HAS-BLED and HAS-BED Versus CHADS$_2$ and CHA$_2$DS$_2$VASC Stroke and Bleeding Scores in Patients With Atrial Fibrillation. *American Journal of Cardiology*. 2017;119(7):1012-6.

10. Retter A, Barrett NA. The management of abnormal haemostasis in the ICU. *Anaesthesia*. 2015;70 Suppl 1:121-7, e40-1.

11. Sibley S, Muscedere J. New-onset atrial fibrillation in critically ill patients. *Can Respir J.*2015;22(3):179-82.

12. Darwish OS, Strube S, Nguyen HM et al. Challenges of anticoagulation for atrial fibrillation in patients with severe sepsis. *Ann Pharmacother*. 2013;47(10):1266-71.

13. Moher D, Liberati A, Tetzlaff J et al. Preferred reporting items for systematic reviews and meta-analyses: the PRISMA statement. *PLoS Med*. 2009;6(7):e1000097.

For peer review only

For peer review only - http://bmjopen.bmj.com/site/about/guidelines.xhtml

14. Hutton B, Salanti G, Caldwell DM et al. The PRISMA extension statement for reporting of systematic reviews incorporating network meta-analyses of health care interventions: checklist and explanations. *Ann Intern Med.* 2015;162(11):777-84.

15. Stang A. Critical evaluation of the Newcastle-Ottawa scale for the assessment of the quality of nonrandomized studies in meta-analyses. *Eur J Epidemiol*. 2010;25(9):603-5.

16. Higgins JPT, Thomas J, Chandler J et al. *Cochrane handbook for systematic reviews of interventions.* 2nd edition. Chichester (UK): John Wiley & Sons, 2019.

17. Balshem H HM, Schunemann HJ, Oxman AD et al. GRADE guidelines: 3. Rating the quality of evidence. *J Clin Epidemiol*. 2011;64:401-6.

18. Shaver CM, Chen W, Janz DR et al. Atrial Fibrillation Is an Independent Predictor of Mortality in Critically Ill Patients. *Crit Care Med*. 2015;43(10):2104-11.

For peer review only

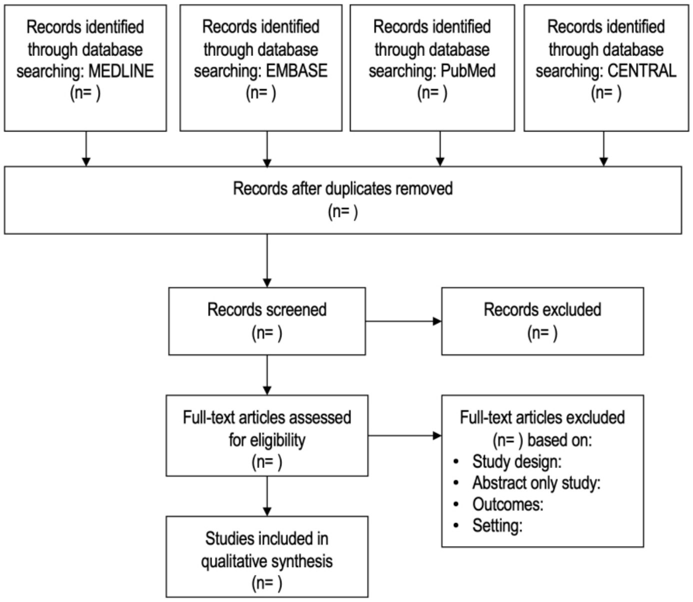

Figure 1 PRISMA flowchart of studies selected in the systematic review.

Figure 1. PRISMA flowchart of studies selected in the systematic review.

209x297mm (300 x 300 DPI)

Supplementary File: Search Strategy used to identify papers included in this review using the Healthcase Databases Advanced Search engine.

DATABASE	MESH TERMS	KEYWORDS (TI AND AB)
MEDLINE	ANTICOAGULANTS/ OR "4HYDROXYCOUMARINS"/ OR ABCIXIMAB/ OR ACENOCOUMAROL/ OR ANCROD/ OR ANTITHROMBINS/ OR BECAPLERMIN/ OR "BETA 2-GLYCOPROTEIN I"/ OR "BLOOD COAGULATION FACTOR INHIBITORS"/ OR "CITRIC ACID"/ OR DALTEPARIN/ OR "DERMATAN SULFATE"/ OR DEXTRANS/ OR DICUMAROL/ OR "EDETIC ACID"/ OR ENOXAPARIN/ OR "ETHYL BISCOUMACETATE"/ OR "FIBRIN FIBRINOGEN DEGRADATION PRODUCTS"/ OR GABEXATE/ OR HEPARIN/ OR "HEPARIN, LOWMOLECULAR-WEIGHT"/ OR HEPARINOIDS/ OR NADROPARIN/ OR "PENTOSAN SULFURIC POLYESTER"/ OR PHENINDIONE/ OR PHENPROCOUMON/ OR "PROTEIN C"/ OR "PROTEIN S"/ OR "SODIUM CITRATE"/ OR TINZAPARIN/ OR WARFARIN/ exp "BLOOD COAGULATION"/	(anticoagul* OR anti-coagul* OR warfarin OR heparin* OR antiplatelet* OR anti-platelet* OR "Direct Oral Anticoagul*" OR "DOAC*" OR "new oral anticoagul*" OR "NOAC*" OR apixaban OR dabigatran OR "dabigatran etexilate" OR rivaroxaban OR "vitamin K antagonist*" OR "VKA").ti,ab
	exp "ATRIAL FIBRILLATION"/	("atrial fibrillation" OR "AF" OR "atrial flutter*" OR "supraventricular tachy*" OR "SVT").ti,ab
	exp "CRITICAL CARE"/	("critical care" OR "Intensive care" OR "ITU" OR "critical* ill*" OR "high depend*" OR "intensive therapy").ti,ab
EMBASE	exp "ANTICOAGULANT AGENT"/	(anticoagul* OR anti-coagul* OR warfarin OR heparin* OR antiplatelet* OR anti-platelet* OR "Direct Oral Anticoagul*" OR "DOAC*" OR "new oral anticoagul*" OR "NOAC*" OR apixaban OR dabigatran OR "dabigatran etexilate" OR rivaroxaban OR "vitamin K antagonist*" OR "VKA").ti,ab
	exp "ATRIAL FIBRILLATION"/	("atrial fibrillation" OR "AF" OR "atrial flutter*" OR "supraventricular tachy*" OR "SVT").ti,ab
	"INTENSIVE CARE"/	("critical care" OR "Intensive care" OR "ITU" OR "critical* ill*" OR "high depend*" OR "intensive therapy").ti,ab
PUBMED		(anticoagul* OR anti-coagul* OR warfarin OR heparin* OR antiplatelet* OR anti-platelet* OR "Direct Oral Anticoagul*" OR "DOAC*" OR "new oral anticoagul*" OR "NOAC*" OR apixaban OR dabigatran OR "dabigatran etexilate" OR rivaroxaban OR "vitamin K antagonist*" OR "VKA").ti,ab
		("atrial fibrillation" OR "AF" OR "atrial flutter*" OR "supraventricular tachy*" OR "SVT").ti,ab
		("critical care" OR "Intensive care" OR "ITU" OR "critical* ill*" OR "high depend*" OR "intensive therapy").ti,ab

For peer review only

COCHRANE		(anticoag*)
		"atrial fibrillation" OR AF
		"Critical care" OR "Intensive care" OR "ITU" OR "high depend*" OR "HDU"

For peer review only

Reporting checklist for protocol of a systematic review.

Based on the PRISMA-P guidelines.

Instructions to authors

Complete this checklist by entering the page numbers from your manuscript where readers will find each of the items listed below.

Your article may not currently address all the items on the checklist. Please modify your text to include the missing information. If you are certain that an item does not apply, please write "n/a" and provide a short explanation.

Upload your completed checklist as an extra file when you submit to a journal.

In your methods section, say that you used the PRISMA-Preporting guidelines, and cite them as:

Moher D, Shamseer L, Clarke M, Ghersi D, Liberati A, Petticrew M, Shekelle P, Stewart LA. Preferred Reporting Items for Systematic Review and Meta-Analysis Protocols (PRISMA-P) 2015 statement. Syst Rev. 2015;4(1):1.

		Reporting Item	Page Number
**Title**			
Identification	#1a	Identify the report as a protocol of a systematic review	1
Update	#1b	If the protocol is for an update of a previous systematic review, identify as such	N/A
**Registration**			
	#2	If registered, provide the name of the registry (such as PROSPERO) and registration number	1
**Authors**			
Contact	#3a	Provide name, institutional affiliation, e-mail address of all protocol authors; provide physical mailing address of corresponding author	1
Contribution	#3b	Describe contributions of protocol authors and identify the guarantor of the review	12
**Amendments**			

For peer review only - http://bmjopen.bmj.com/site/about/guidelines.xhtml

	#4	If the protocol represents an amendment of a previously completed or published protocol, identify as such and list changes; otherwise, state plan for documenting important protocol amendments	N/A
**Support**			
Sources	#5a	Indicate sources of financial or other support for the review	12
Sponsor	#5b	Provide name for the review funder and / or sponsor	N/A
Role of sponsor or funder	#5c	Describe roles of funder(s), sponsor(s), and / or institution(s), if any, in developing the protocol	N/A
**Introduction**			
Rationale	#6	Describe the rationale for the review in the context of what is already known	3,4,5
Objectives	#7	Provide an explicit statement of the question(s) the review will address with reference to participants, interventions, comparators, and outcomes (PICO)	5
**Methods**			
Eligibility criteria	#8	Specify the study characteristics (such as PICO, study design, setting, time frame) and report characteristics (such as years considered, language, publication status) to be used as criteria for eligibility for the review	5,6,7
Information sources	#9	Describe all intended information sources (such as electronic databases, contact with study authors, trial registers or other grey literature sources) with planned dates of coverage	8,9
Search strategy	#10	Present draft of search strategy to be used for at least one electronic database, including planned limits, such that it could be repeated	13
Study records - data management	#11a	Describe the mechanism(s) that will be used to manage records and data throughout the review	9
Study records - selection process	#11b	State the process that will be used for selecting studies (such as two independent reviewers) through each phase of the review (that is, screening, eligibility and inclusion in meta-analysis)	9

For peer review only

Study records - data collection process	#11c	Describe planned method of extracting data from reports (such as piloting forms, done independently, in duplicate), any processes for obtaining and confirming data from investigators	9
Data items	#12	List and define all variables for which data will be sought (such as PICO items, funding sources), any pre-planned data assumptions and simplifications	10
Outcomes and prioritization	#13	List and define all outcomes for which data will be sought, including prioritization of main and additional outcomes, with rationale	7,8
Risk of bias in individual studies	#14	Describe anticipated methods for assessing risk of bias of individual studies, including whether this will be done at the outcome or study level, or both; state how this information will be used in data synthesis	10
Data synthesis	#15a	Describe criteria under which study data will be quantitatively synthesised	10,11
Data synthesis	#15b	If data are appropriate for quantitative synthesis, describe planned summary measures, methods of handling data and methods of combining data from studies, including any planned exploration of consistency (such as I2, Kendall's τ)	11
Data synthesis	#15c	Describe any proposed additional analyses (such as sensitivity or subgroup analyses, meta-regression)	11,12
Data synthesis	#15d	If quantitative synthesis is not appropriate, describe the type of summary planned	11
Meta-bias(es)	#16	Specify any planned assessment of meta-bias(es) (such as publication bias across studies, selective reporting within studies)	N/A
Confidence in cumulative evidence	#17	Describe how the strength of the body of evidence will be assessed (such as GRADE)	11,12

The PRISMA-P checklist is distributed under the terms of the Creative Commons Attribution License CC-BY 4.0. This checklist was completed on 08. February 2020 using https://www.goodreports.org/, a tool made by the EQUATOR Network in collaboration with Penelope.ai

For peer review only

BMJ Open

Anticoagulation strategies in critical care for the treatment of atrial fibrillation: a protocol for a systematic review and meta-analysis

Journal:	*BMJ Open*
Manuscript ID	bmjopen-2020-037591.R1
Article Type:	Protocol
Date Submitted by the Author:	14-Jul-2020
Complete List of Authors:	Johnston, Brian; University of Liverpool Faculty of Health and Life Sciences,<br>Nelson, Alexandra; University of Liverpool Faculty of Health and Life Sciences<br>Waite, Alicia; University of Liverpool Institute of Ageing and Chronic Disease<br>Lemma, Gedeon; University of Liverpool Faculty of Health and Life Sciences<br>Welters, Ingeborg; University of Liverpool Institute of Ageing and Chronic Disease,
**Primary Subject Heading**:	Intensive care
Secondary Subject Heading:	Cardiovascular medicine, Anaesthesia, Pharmacology and therapeutics, Haematology (incl blood transfusion), Evidence based practice
Keywords:	INTENSIVE & CRITICAL CARE, Adult intensive & critical care < ANAESTHETICS, CARDIOLOGY, Anticoagulation < HAEMATOLOGY

SCHOLARONE™
Manuscripts

BMJ

I, the Submitting Author has the right to grant and does grant on behalf of all authors of the Work (as defined in the below author licence), an exclusive licence and/or a non-exclusive licence for contributions from authors who are: i) UK Crown employees; ii) where BMJ has agreed a CC-BY licence shall apply, and/or iii) in accordance with the terms applicable for US Federal Government officers or employees acting as part of their official duties; on a worldwide, perpetual, irrevocable, royalty-free basis to BMJ Publishing Group Ltd ("BMJ") its licensees and where the relevant Journal is co-owned by BMJ to the co-owners of the Journal, to publish the Work in this journal and any other BMJ products and to exploit all rights, as set out in our licence.

The Submitting Author accepts and understands that any supply made under these terms is made by BMJ to the Submitting Author unless you are acting as an employee on behalf of your employer or a postgraduate student of an affiliated institution which is paying any applicable article publishing charge ("APC") for Open Access articles. Where the Submitting Author wishes to make the Work available on an Open Access basis (and intends to pay the relevant APC), the terms of reuse of such Open Access shall be governed by a Creative Commons licence – details of these licences and which Creative Commons licence will apply to this Work are set out in our licence referred to above.

Other than as permitted in any relevant BMJ Author's Self Archiving Policies, I confirm this Work has not been accepted for publication elsewhere, is not being considered for publication elsewhere and does not duplicate material already published. I confirm all authors consent to publication of this Work and authorise the granting of this licence.

For peer review only

Anticoagulation strategies in critical care for the treatment of atrial fibrillation: a protocol for a systematic review and meta-analysis

Authors

1) Brian W Johnston[1,2], 2) Alexandra Nelson[2], 3) Alicia C Waite[1,2], 4) Gedeon Lemma[2], 5) Ingeborg D Welters[1,2]

Corresponding author: Brian W Johnston, c/o Intensive Care Unit, Royal Liverpool and Broadgreen University Hospital, Prescott Street, L78XP, Contact Telephone: 0151 706 2410

Author Affiliations

1. University of Liverpool and The Royal Liverpool University Hospital, Liverpool Healthcare Partners, Liverpool, UK.
2. The University of Liverpool, Liverpool, UK.

Author contact details

1. Dr Brian. W Johnston, brian.johnston@liverpool.ac.uk, ORCID: 0000-0003-1634-3297
2. Miss Alexandra Nelson, hlanels2@liverpool.ac.uk
3. Dr Alicia C Waite, Alicia.Waite@liverpool.ac.uk, ORCID: 0000-0001-8734-994X
4. Mr Gedeon Lemma, hlglemma@liverpool.ac.uk
5. Prof Ingeborg D Welters, Ingeborg.welters@liverpool.ac.uk, ORCID: 0000-0002-3408-8798

Keywords

Atrial Fibrillation, Critical Illness, Intensive Care Unit, Anticoagulation,

Prospero Trials Registration Number: CRD42020158237

For peer review only

ABSTRACT

Introduction

Atrial fibrillation is the most common cardiac arrhythmia in critically ill patients and is associated with an increased risk of thromboembolic events and mortality. Oral anticoagulation for thromboembolism prophylaxis is a key component of managing atrial fibrillation in the general population and is recommended by NICE guidelines. However, assessment tools used to aid decision making about anticoagulation have not yet been validated in the critical care setting. There is a paucity of data assessing the impact of anticoagulation strategies on clinical outcomes in critically ill patients with atrial fibrillation. We present a protocol for a systematic review and meta-analysis to evaluate the effectiveness of anticoagulation strategies for atrial fibrillation used specifically in critical care.

Methods and analysis

We will conduct a systematic review of the literature by searching MEDLINE, EMBASE, CENTRAL and PubMed databases for articles published from January 1990 to October 2019. Studies reporting anticoagulation strategies for AF in adults (>18 years) admitted to a general critical care setting will be assessed for inclusion. Outcomes of interest will include, 1) percentage of patients commenced on anticoagulation in critical care for atrial fibrillation, 2) incidence of thromboembolism, 3) incidence of bleeding events, 4) ICU mortality, 5) hospital mortality, 6) ICU length of stay and 7) hospital length of stay. We will conduct a meta-analysis of trials. Risk of bias will be assessed using the Cochrane Risk of Bias tool for randomised trials or the Newcastle-Ottowa Risk of Bias assessment tool for non-randomised studies. This protocol and subsequent systematic review will be reported following the Preferred Reporting Items for Systematic Reviews and Meta-Analyses checklist.

Ethics and dissemination

This proposed systematic review will include data extracted from published studies, therefore ethical approval is not required. The results of this review will be published in clinical specialty journals and presented at international meetings and conferences.

Prospero Trial Registration Number: CRD42020158237

For peer review only

Strengths and limitations of this study

Strengths:

- This systematic review follows the Preferred Reporting Items for Systematic Review and Meta-Analysis Protocols guidelines.
- Study selection, data extraction and risk of bias assessment will be performed independently by two researchers, ensuring all relevant studies are included without personal biases.
- There are no language publication restrictions to this systematic review eliminating language bias.

Limitations:

- Non-randomised studies will be included in this review potentiating a high risk of bias in included studies.
- Given the particular risk for thromboembolic events in critically ill patients, we will only include studies on patients receiving anticoagulation during their critical care admission.

INTRODUCTION

Background

Atrial fibrillation (AF) is the most common cardiac arrhythmia in the critical care population. AF is characterised by erratic conduction of electrical impulses and uncoordinated contraction of the atria which ultimately increases the risk of heart failure, stroke and death [1,2]. Nearly one third of patients admitted to critical care have a diagnosis of pre-existing atrial fibrillation (PEAF), however up to two thirds develop new-onset atrial fibrillation (NOAF) during their admission [2]. AF can trigger rapid ventricular rates resulting in haemodynamic compromise and a loss of cardiac output. This may contribute to acute decompensation of already unstable critically ill patients and eventually lead to the increased morbidity and mortality associated with AF in critical care [2,3].

In addition to the traditional risk factors for the development of AF, including advanced age, hypertension, ischaemic heart disease, heart failure and valvular disease; there are factors related to being critically ill that predispose patients to the development of NOAF [1,2]. These factors include electrolyte abnormalities,

For peer review only

hypoxaemia, adrenergic overstimulation, progressive autonomic dysfunction, acute systemic inflammation, sepsis and shock [1,2]. Changes in autonomic activity resulting from vasopressor administration as well as electrolyte disturbances are frequent among critically ill patients and can lead to increased atrial ectopic impulses and subsequent NOAF [3]. There is also evidence that central venous catheters induce mechanical irritation of the atria and may be a contributing factor in the development of NOAF [1]. The high incidence of NOAF in critical care is likely due to a combination of these traditional and critical illness specific related risk factors [3].

Both PEAF and NOAF are associated with a myriad of complications in critical care. As a result of inefficient atrial systole, the reduced blood flow velocity in the left atria predisposes patients with AF to cardiac and systemic emboli [4]. Embolic events, such as ischaemic stroke, are common and disabling complications of AF and cause a significant disease burden [5]. AF in the critical care setting is associated with a twofold increased risk of stroke and a two to fivefold increased risk of mortality [2,6].

Oral anticoagulation for thromboembolism prophylaxis is a key component of managing AF in the general population [7]. In the United Kingdom, clinicians are guided in the management of NOAF by the recommendations from the National Institute for Health and Care Excellence (NICE). With regards to anticoagulation for NOAF, NICE recommends the use of validated tools assessing thromboembolic risk (e.g. CHA_2DS_2-VASc) and bleeding risk (e.g. HASBLED) to stratify patients that may benefit from systemic anticoagulation through prevention of thromboembolic events such as stroke [7]. However, the risk-benefit tools used to aid decision making about anticoagulation, such as the CHA_2DS_2-VASc and HASBLED scores, have not been validated in critical care populations [8,9]. Decisions around anticoagulation strategies in critical care are complex and challenging. Critically unwell patients are at a significantly increased risk of bleeding, but may also of hypercoagulable, due to the abnormal haemostasis that is associated with critical illness [9]. Factors contributing to the acquired coagulopathy in critical care include severe sepsis, thrombocytopenia, haemodilution of clotting factors following blood transfusion, disseminated intravascular coagulation (DIC), acute kidney injury (AKI) and liver failure [10]. The potential need for urgent procedures or insertion of invasive devices, such as arterial lines and central venous catheters, poses an additional challenge in

For peer review only - http://bmjopen.bmj.com/site/about/guidelines.xhtml

effectively anticoagulating these patients [11]. Combined with the added effect of anticoagulation itself, the high risk of bleeding may preclude safe therapeutic anticoagulation for AF in critical care patients. Guidelines from NICE recommend anticoagulation with Heparin in patients presenting with NOAF that are receiving no or subtherapeutic anticoagulation. Furthermore, they recommend that patients who have failed to achieve stable sinus rhythm within 48 hours or have risk factors for the recurrence of NOAF should be offered oral anticoagulation long term. Despite these recommendations a nationwide survey of intensive care clinicians revealed that 63.8% of clinicians would not routinely anticoagulate critically ill patients with NOAF while 30.8% would consider anticoagulation if NOAF persisted beyond 72 hours rather than the recommended 48 hours by NICE [12]. Furthermore, 98% of critical care clinicians revealed that they would be happy to administer Heparin as anticoagulation but not an oral anticoagulation [12]. The variation in practice likely represents the unique challenges of managing NOAF in critically unwell patients, in which administration of oral anticoagulants may not possible in sedated patients or via nasogastric tube and highlights the need for guidelines specific to critically ill patients.

Why is it important to undertake this review?

There is a paucity of data assessing the impact of anticoagulation strategies on clinical outcomes in critically unwell patients with AF [13]. A consensus on an effective anticoagulation strategy for thromboembolism prophylaxis has not yet been reached and current recommendations regarding anticoagulation therapy are largely based on observational studies and expert opinion [3]. We therefore have designed a protocol for a systematic review and meta-analysis of the literature around the anticoagulation for AF in the critically unwell and critical care setting. We will assess the existing literature to define anticoagulation strategies in critical care for both NOAF and PEAF. We anticipate that the results of this review will highlight areas were evidence is lacking, trigger further research and contribute to the development of new guidelines specific to the management of anticoagulation in patients with AF in the critical care setting.

Objective

To conduct a systematic review and meta-analysis of the literature to determine the use and effectiveness of anticoagulation strategies for AF in critical care and identify the associated risks and benefits of therapeutic anticoagulation for AF.

For peer review only - http://bmjopen.bmj.com/site/about/guidelines.xhtml

METHODS AND ANALYSIS

This systematic review will be conducted in accordance with The Cochrane Collaboration principles of Systematic Reviews and will be reported following the Preferred Reporting Items for Systematic Reviews and Meta-Analyses (PRISMA) guidelines [14,15].

This protocol has been registered with the International Prospective Register of Systematic Reviews (PROSPERO) database. PROSPERO registration number: CRD42020158237

Inclusion criteria

Type of studies

All quantitative studies that report anticoagulation strategies for AF, in an adult critical care setting will be assessed for inclusion. Eligible studies will include randomised and non-randomised studies.

Eligible studies must include both a cohort of patients with AF who were anticoagulated and a cohort of patients with AF who were not anticoagulated. We will include studies in patients diagnosed with NOAF (including paroxysmal AF) or PEAF (including permanent AF) by rhythm classification by continuous ECG monitoring or 12 lead ECG.

We will include studies conducted in level 2 and level 3 critical care settings as defined by Marshall et al [16]. We will include studies enrolling patients from general medical, general surgical or mixed mixed/surgical patients.

Phenomenon of interest

Studies must include patients who have been in AF for >48 hours, based on the NICE guidelines on initiating anticoagulant treatment [7], and may include both PEAF and NOAF. Studies must describe outcomes associated with the chosen anticoagulation strategy including the outcomes of interest described below.

For peer review only

Population

Studies that include adult patients admitted to the ICU types specified above will be assessed for inclusion. For the purpose of this review, an adult is defined as ≥18 years.

Type of intervention and comparator(s)

Interventions:

Eligible studies will include any of the following treatments:

1) Warfarin
2) Any other vitamin K antagonist
3) Unfractionated heparin (UFH)
4) Treatment dose low molecular weight heparin (LMWH)
5) Factor Xa inhibitors (e.g Edoxaban, Apixaban, Rivaroxaban)
6) Direct thrombin (Factor IIa) inhibitors (Dabigatran, Inogatran, Melagatran, Argatroban)
7) Any combination of the above interventions

Comparators:

Comparators will include:

1) Any of the interventions above
2) Placebo
3) Standard care where it does not include anticoagulation

Exclusion criteria

Types of studies

Qualitative studies, case studies, editorials, letters, abstract only reports, reviews and commentaries that do not include original information will be excluded from this review.

For peer review only

Studies of cohorts that have undergone or plan to undergo cardiothoracic surgery, permanent pacemaker insertion or surgical ablation will be excluded. Studies based on service-specific ICUs, for example cardiac, cardiothoracic surgical or neurosurgical units, will be excluded in addition to studies based on acute medical units or in emergency departments.

Phenomenon of interest

Studies will be excluded if patients have been commenced on any anticoagulation therapy for a reason other than AF and cannot be disaggregated from the entire cohort. Studies that include patients with an inherited or pre-existing bleeding disorder or clotting disorder will also be excluded.

Population

Studies of participants under 18 years, pregnant women and patients with clear contraindication to anticoagulation therapy for example intracranial haemorrhage, overt bleeding or allergy to anticoagulant medication will be excluded.

Outcome measures

Primary outcome measure:

1) Percentage of patients commenced on anticoagulation for AF (NOAF and/or PEAF) in a critical care setting including but not limited to warfarin, LMWH, UFH, Factor Xa inhibitors, Factor IIa inhibitors.

Secondary outcome measures:

1) Incidence of thromboembolic events (defined as stroke, mesenteric ischaemia, acute limb ischaemia, pulmonary embolism) during critical care admission to identify the outcomes of anticoagulation therapy in the critical care setting.
2) Incidence of any in hospital thromboembolic events (defined as stroke, mesenteric ischaemia, acute limb ischaemia, pulmonary embolism).
3) Development of a major haemorrhage event (defined clinically as haemorrhage leading to death, signs of shock, requirement for blood transfusion or urgent endoscopic / surgical intervention) [17].

For peer review only

For peer review only - http://bmjopen.bmj.com/site/about/guidelines.xhtml

4) ICU length of stay (LOS)
5) ICU mortality
6) 28-day mortality
7) 90-day mortality
8) 365-day mortality
9) Use of risk stratification scores for anticoagulation decision making (such as CHA_2DS_2-VASc and HASBLED)
10) Severity scores of critical illness (e. g. APACHE II and SOFA)

Search strategy

We will engage the services of a health information specialist, and a comprehensive broad literature search will be conducted. Medical subject headings will be used to identify papers that matched the search index and relevant selected free text terms will also be used.

The search strategies identified will focus on the population (critically ill), the participants (patients with AF) and the intervention (any anticoagulation strategy mentioned previously). The search strategy will not be limited by outcomes studied in order to broaden the scope of eligible papers.

Database searches

We will search the following databases for studies published between January 1990 and October 2019;

1) MEDLINE Ovid, Medline Ovid Epub Ahead of Print, and Medline Ovid In-Process
2) EMBASE Ovid
3) The Cochrane Central Register of Controlled Trials
4) PUBMED
5) ClinicalTrials.gov

For peer review only

MEDLINE, EMBASE and PUBMED will be access via NICE Healthcare Database Advanced Search (HDAS) using OpenAthens. We will screen referenced papers following full text screening and any review articles identified during the screening process. We will screen for relevant conference proceedings and include any potentially relevant studies. A full description of the search strategy that will be used in HDAS (supplementary file).

Citation management and screening

Results from searches in all databases will be exported to Endnote X9 (Clarivate analytics) and duplicates will be removed. All citations will be imported into the Covidence systematic review platform (Veritas Health Innovation, Melbourne, Australia) [18]. Titles and abstracts will be screened in duplicate by two independent reviewers for relevance to the review and eliminated as appropriate. Full text articles that are not excluded after title and abstract screening, will be screened against the inclusion and exclusion criteria by two independent reviewers. Any discrepancies or conflicts in the screening process will be resolved by discussion and subsequent input of a third reviewer. Primary reason for exclusion will be documented in Covidence and the screening process will be documented in a PRISMA flowchart (Figure 1).

Data extraction and management

Data will be extracted in duplicate by two independent reviewers using a standardised, pre-piloted data extraction form.

Information, including the following characteristics, will be extracted from studies:

1) Study design and methodology, including title, authors, journal, publication date, study type, study period and number of participants
2) All inclusion criteria previously mentioned
3) Population characteristics, including age, sex, ICU admission diagnosis, location, co-morbidities, illness severity scores
4) Recruitment procedures
5) Interventions

For peer review only

For peer review only - http://bmjopen.bmj.com/site/about/guidelines.xhtml

6) The primary and secondary outcome measures and reported findings of the study

Any discrepancies within data extraction will be resolved through discussion and consultation with a third reviewer. We will contact authors if clarification regarding the data or methodology is required. If data cannot be obtained from the authors, the impact will be discussed as a limitation of the review.

Risk of bias assessment

Risk of bias will be assessed using a modified Newcastle-Ottawa Scale (mNOS), a scoring system for non-randomised trials [19]. mNOS uses a system by which the study is judged on three broad perspectives: the selection of the study groups, the comparability and the ascertainment of outcome of interest.

Included randomised controlled trials will be assessed with the revised Cochrane Collaborations Risk of Bias (RoB 2) tool using the criteria outlined in the Cochrane Handbook of Systematic Reviews of Interventions [20]. The RoB 2 tool assesses the following domains: selection, comparability, outcome performance and will be presented in table format.

Data Synthesis and Analysis

A description of included studies will be reported in evidence tables and discussed in the text. We will report participant characteristics, interventions, clinical outcomes and methodological quality.

Primary outcomes will be reported as the percentage of patients that received anticoagulation for any AF (NOAF and PEAF) in critical care. If sufficient data allows, we will report the percentage of patients commenced on anticoagulation according to type of AF (NOAF and/or PEAF).

We anticipate that the majority of studies included in our systematic review and meta-analysis will be case control studies. Therefore, we will present dichotomous data as number of participants experiencing the outcome with OR and 95% confidence intervals [21]. For continuous outcome data, we will extract arithmetic means and standard deviations with 95% confidence intervals for each outcome, together with the numbers analysed in each group. We will also extract medians and ranges where provided.

For peer review only

Data from individual studies will be pooled and presented visually by Forest plot. We anticipate studies to vary significantly, therefore we will undertake a meta-analysis of included studies by using a random effects model with 95% CI to estimate pooled estimates of effect. We will assess for heterogeneity between studies using the Cochran's Q test and report the degree of heterogeneity using the I^2 statistic in RevMan (Review Manager, 2014). Heterogeneity will be considered low if values are below 25%, moderated if values are between 25% and 75% and high if values are above 75%. Suitability for meta-analysis will be determined by the degree of clinical, statistical and methodological heterogeneity observed between studies. We will explore heterogeneity between studies by subgroup analysis assessing a number of factors such as study design, intervention and participants. Where there is a high degree of heterogeneity (>75%) that cannot be explained by clinical or methodological variation, we will not undertake a meta-analysis of results. If we are unable to pool studies due to heterogeneity, we will describe the findings via a narrative synthesis. Meta-analysis will be conducted using the Cochrane Collaborations RevMan (Review Manager, 2014) software.

We will assess the certainty of our evidence for each outcome according to the Grading of Recommendations Assessment, Development and Evaluation (GRADE) approach [22]. GRADE methodology assesses the certainty in evidence across the domains of risk of bias, consistency, directness, precision and publication bias. The certainty of evidence will be reported as high, moderate, low or very low.

Subgroup analysis

If we extract sufficient data, we will undertake subgroup analysis, assessing the following:

1. Type of anticoagulant therapy
2. NOAF versus PEAF
3. Sepsis versus non-sepsis
4. Illness severity scores (APACHE II score, SOFA score)
5. Risk scores for bleeding and thromboembolic events (HASBLED, CHA_2DS_2-VASc)
6. Medical versus surgical ICU admission diagnosis

For peer review only

Patient and public involvement

This protocol will use previously published data. As such there will be no patient and public involvement in the design of the study, interpretation of the results. We will disseminate the results of this review to any interested patients and it will be freely accessible in open access journals.

Ethics and dissemination

This proposed systematic review will not include primary data and will extract data from published studies, therefore ethical approval is not required. The results of this review will be disseminated through publication in clinical specialty journals, including open access journals, and various medias including international meetings, conferences, congresses and symposiums.

Discussion

This systematic review will assess the evidence available for anticoagulation strategies used in the management of AF in critically ill patients. AF affects up to 25% of critical care admissions, hence the need for systematic evaluation of anticoagulation strategies used in critical care and their impact on patient outcomes [23]. AF is associated with an increased risk of thromboembolic complications, but scoring systems used to aid decision making on whether and how-to anticoagulate patients with AF have not been validated for use in the critical care setting. This review will enable a comparison of the anticoagulation strategies used for both PEAF and NOAF in critical care and aims to assess outcomes including thromboembolic events, haemorrhagic events, length of stay (LOS) in critical care and mortality on ICU. Critically ill patients have a particularly high risk of thromboembolic events, which could theoretically be ameliorated by administering anticoagulant therapy during critical illness. A limitation of our protocol is that results will not be generalizable to patients who are clinically stable after having been critically ill.

Based on a preliminary search of the literature, we believe that this is the first systematic review to consider anticoagulation strategies used in the treatment of AF in the critically ill patient cohort. We aim for this review to provide an evidence base upon which recommendations can be made, to ultimately reduce the impact of

For peer review only

For peer review only - http://bmjopen.bmj.com/site/about/guidelines.xhtml

AF on patient outcomes during and after critical care admission. We will publish the results of this review in clinical specialty journals and we present it at international meetings and conferences. We will also make our report available via our web- based repository and update our PROSPERO entry with the web address details.

FOOTNOTES

Author's contribution

The protocol was conceived and designed by IW, BWJ and AW. AN will conduct primary screening and data collection, reviewed by BWJ and GL. Data extraction, analysis and preparation of the manuscript will be conducted by AN and BWJ. IW, BWJ, AW, GL and AN will read and approve the final manuscript.

Funding

This research received no specific grant from any funding agency in the public, commercial or not-for-profit sectors.

Conflicts of interest

None declared.

Data Sharing Statement

All data is included in the manuscript.

Provenance and peer review

Not commissioned; externally peer reviewed.

Patient consent

Not required.

Patient and public involvement

There was no patient or public involvement in the planning or writing of this protocol

Figure Legend

Figure 1: PRISMA flowchart of studies selected in the systematic review

For peer review only

REFERENCES

1. Malik A, Candilio L, Hausenloy DJ. Atrial fibrillation in the intensive care setting. *Journal of the Intensive Care Society.* 2013;14(2):141-9.

2. Bosch NA, Cimini J, Walkey AJ. Atrial Fibrillation in the ICU. *Chest*. 2018;154(6):1424-34.

3. Walkey AJ, Hogarth DK, Lip GYH. Optimizing Atrial fibrillation management from ICU and beyond. *Chest*. 2015;148(4):859-64.

4. Nadzeya Kuzniatsova, Lip GYH. Prevention of Thromboembolism in Atrial Fibrillation Patients. *European Cardiology Review*. 2011;7(1):37-43.

5. Gorczyca I, Wozakowska-Kaplon B, Starzyk K et al. Evaluation of the recommended prevention of thrombosis in hospitalised patients with atrial fibrillation and high thromboembolism risk. *Kardiol Pol.* 2018;76(3):625-32.

6. Arrigo M, Bettex D, Rudiger A. Management of atrial fibrillation in critically ill patients. *Crit Care Res Pract*. 2014;2014:840615-.

7. Gilligan DM, Ellenbogen K, Epstein E. Atrial Fibrillation: Management 2014 [updated August 2014.] Available from: https://www.nice.org.uk/guidance/cg180

8. Kanji S, Williamson DR, Yaghchi BM et al. Epidemiology and management of atrial fibrillation in medical and noncardiac surgical adult intensive care unit patients. *J Crit Care.* 2012;27(3):326.e1-8.

9. Poli D, Antonucci E, Pengo V et al. Comparison of HAS-BLED and HAS-BED Versus $CHADS_2$ and CHA_2DS_2VASC Stroke and Bleeding Scores in Patients With Atrial Fibrillation. *American Journal of Cardiology*. 2017;119(7):1012-6.

10. Retter A, Barrett NA. The management of abnormal haemostasis in the ICU. *Anaesthesia*. 2015;70 Suppl 1:121-7, e40-1.

11. Sibley S, Muscedere J. New-onset atrial fibrillation in critically ill patients. *Can Respir J.*2015;22(3):179-82.

12. Chean CS, McAuley D, Gordon A, Welters ID. Current practice in the management of new-onset atrial fibrillation in critically ill patients: a UK-wide survey. PeerJ. 2017;5:e3716.

13. Darwish OS, Strube S, Nguyen HM et al. Challenges of anticoagulation for atrial fibrillation in patients with severe sepsis. *Ann Pharmacother*. 2013;47(10):1266-71.

For peer review only

14. Moher D, Liberati A, Tetzlaff J et al. Preferred reporting items for systematic reviews and meta-analyses: the PRISMA statement. *PLoS Med*. 2009;6(7):e1000097.

15. Hutton B, Salanti G, Caldwell DM et al. The PRISMA extension statement for reporting of systematic reviews incorporating network meta-analyses of health care interventions: checklist and explanations. *Ann Intern Med.* 2015;162(11):777-84.

16. Marshall JC, Bosco L, Adhikari NK, Connolly B, Diaz JV, Dorman T, et al. What is an intensive care unit? A report of the task force of the World Federation of Societies of Intensive and Critical Care Medicine. J Crit Care. 2017;37:270-6.

17. Roberts I, Shakur-Still H, Afolabi A, Akere A, Arribas M, Brenner A, et al. Effects of a high-dose 24-h infusion of tranexamic acid on death and thromboembolic events in patients with acute gastrointestinal bleeding (HALT-IT): an international randomised, double-blind, placebo-controlled trial. The Lancet. 2020;395(10241):1927-36.

18. Covidence systematic review software, Veritas Health Innovation, Melbourne, Australia. Available at www.covidence.org

19. Stang A. Critical evaluation of the Newcastle-Ottawa scale for the assessment of the quality of nonrandomized studies in meta-analyses. *Eur J Epidemiol*. 2010;25(9):603-5.

20. Higgins JPT, Thomas J, Chandler J et al. *Cochrane handbook for systematic reviews of interventions.* 2nd edition. Chichester (UK): John Wiley & Sons, 2019.

21. Higgins JPT, Li T, Deeks JJ. Chapter 6: Choosing effect measures and computing estimates of effect. In: Higgins JPT, Thomas J, Chandler J, Cumpston M, Li T, Page MJ, Welch VA (editors). *Cochrane Handbook for Systematic Reviews of Interventions* version 6.0 (updated July 2019). Cochrane, 2019. Available from www.training.cochrane.org/handbook.

22. Balshem H HM, Schunemann HJ, Oxman AD et al. GRADE guidelines: 3. Rating the quality of evidence. *J Clin Epidemiol*. 2011;64:401-6.

23. Shaver CM, Chen W, Janz DR et al. Atrial Fibrillation Is an Independent Predictor of Mortality in Critically Ill Patients. *Crit Care Med*. 2015;43(10):2104-11.

For peer review only

For peer review only - http://bmjopen.bmj.com/site/about/guidelines.xhtml

For peer review only

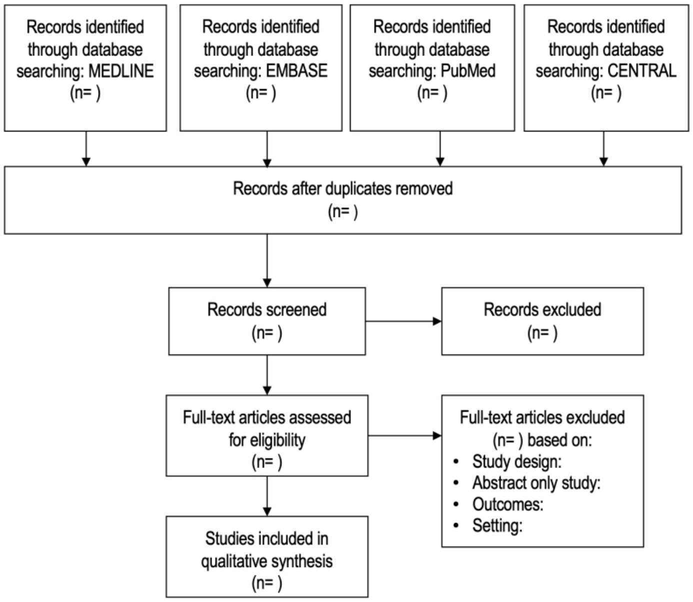

Figure 1 PRISMA flowchart of studies selected in the systematic review.

Figure 1. PRISMA flowchart of studies selected in the systematic review.

209x297mm (300 x 300 DPI)

Supplementary File: Search Strategy used to identify papers included in this review using the Healthcase Databases Advanced Search engine.

DATABASE	MESH TERMS	KEYWORDS (TI AND AB)
MEDLINE	ANTICOAGULANTS/ OR "4HYDROXYCOUMARINS"/ OR ABCIXIMAB/ OR ACENOCOUMAROL/ OR ANCROD/ OR ANTITHROMBINS/ OR BECAPLERMIN/ OR "BETA 2-GLYCOPROTEIN I"/ OR "BLOOD COAGULATION FACTOR INHIBITORS"/ OR "CITRIC ACID"/ OR DALTEPARIN/ OR "DERMATAN SULFATE"/ OR DEXTRANS/ OR DICUMAROL/ OR "EDETIC ACID"/ OR ENOXAPARIN/ OR "ETHYL BISCOUMACETATE"/ OR "FIBRIN FIBRINOGEN DEGRADATION PRODUCTS"/ OR GABEXATE/ OR HEPARIN/ OR "HEPARIN, LOWMOLECULAR-WEIGHT"/ OR HEPARINOIDS/ OR NADROPARIN/ OR "PENTOSAN SULFURIC POLYESTER"/ OR PHENINDIONE/ OR PHENPROCOUMON/ OR "PROTEIN C"/ OR "PROTEIN S"/ OR "SODIUM CITRATE"/ OR TINZAPARIN/ OR WARFARIN/ exp "BLOOD COAGULATION"/	(anticoagul* OR anti-coagul* OR warfarin OR heparin* OR antiplatelet* OR anti-platelet* OR "Direct Oral Anticoagul*" OR "DOAC*" OR "new oral anticoagul*" OR "NOAC*" OR apixaban OR dabigatran OR "dabigatran etexilate" OR rivaroxaban OR "vitamin K antagonist*" OR "VKA").ti,ab
	exp "ATRIAL FIBRILLATION"/	("atrial fibrillation" OR "AF" OR "atrial flutter*" OR "supraventricular tachy*" OR "SVT").ti,ab
	exp "CRITICAL CARE"/	("critical care" OR "Intensive care" OR "ITU" OR "critical* ill*" OR "high depend*" OR "intensive therapy").ti,ab
EMBASE	exp "ANTICOAGULANT AGENT"/	(anticoagul* OR anti-coagul* OR warfarin OR heparin* OR antiplatelet* OR anti-platelet* OR "Direct Oral Anticoagul*" OR "DOAC*" OR "new oral anticoagul*" OR "NOAC*" OR apixaban OR dabigatran OR "dabigatran etexilate" OR rivaroxaban OR "vitamin K antagonist*" OR "VKA").ti,ab
	exp "ATRIAL FIBRILLATION"/	("atrial fibrillation" OR "AF" OR "atrial flutter*" OR "supraventricular tachy*" OR "SVT").ti,ab
	"INTENSIVE CARE"/	("critical care" OR "Intensive care" OR "ITU" OR "critical* ill*" OR "high depend*" OR "intensive therapy").ti,ab
PUBMED		(anticoagul* OR anti-coagul* OR warfarin OR heparin* OR antiplatelet* OR anti-platelet* OR "Direct Oral Anticoagul*" OR "DOAC*" OR "new oral anticoagul*" OR "NOAC*" OR apixaban OR dabigatran OR "dabigatran etexilate" OR rivaroxaban OR "vitamin K antagonist*" OR "VKA").ti,ab
		("atrial fibrillation" OR "AF" OR "atrial flutter*" OR "supraventricular tachy*" OR "SVT").ti,ab
		("critical care" OR "Intensive care" OR "ITU" OR "critical* ill*" OR "high depend*" OR "intensive therapy").ti,ab

For peer review only

COCHRANE		(anticoag*)
		"atrial fibrillation" OR AF
		"Critical care" OR "Intensive care" OR "ITU" OR "high depend*" OR "HDU"

For peer review only

Reporting checklist for protocol of a systematic review.

Based on the PRISMA-P guidelines.

Instructions to authors

Complete this checklist by entering the page numbers from your manuscript where readers will find each of the items listed below.

Your article may not currently address all the items on the checklist. Please modify your text to include the missing information. If you are certain that an item does not apply, please write "n/a" and provide a short explanation.

Upload your completed checklist as an extra file when you submit to a journal.

In your methods section, say that you used the PRISMA-Preporting guidelines, and cite them as:

Moher D, Shamseer L, Clarke M, Ghersi D, Liberati A, Petticrew M, Shekelle P, Stewart LA. Preferred Reporting Items for Systematic Review and Meta-Analysis Protocols (PRISMA-P) 2015 statement. Syst Rev. 2015;4(1):1.

		Reporting Item	Page Number
**Title**			
Identification	#1a	Identify the report as a protocol of a systematic review	1
Update	#1b	If the protocol is for an update of a previous systematic review, identify as such	N/A
**Registration**			
	#2	If registered, provide the name of the registry (such as PROSPERO) and registration number	1
**Authors**			
Contact	#3a	Provide name, institutional affiliation, e-mail address of all protocol authors; provide physical mailing address of corresponding author	1
Contribution	#3b	Describe contributions of protocol authors and identify the guarantor of the review	14

For peer review only

**Amendments**			
	#4	If the protocol represents an amendment of a previously completed or published protocol, identify as such and list changes; otherwise, state plan for documenting important protocol amendments	N/A
**Support**			
Sources	#5a	Indicate sources of financial or other support for the review	14
Sponsor	#5b	Provide name for the review funder and / or sponsor	N/A
Role of sponsor or funder	#5c	Describe roles of funder(s), sponsor(s), and / or institution(s), if any, in developing the protocol	N/A
**Introduction**			
Rationale	#6	Describe the rationale for the review in the context of what is already known	3,4,5
Objectives	#7	Provide an explicit statement of the question(s) the review will address with reference to participants, interventions, comparators, and outcomes (PICO)	5
**Methods**			
Eligibility criteria	#8	Specify the study characteristics (such as PICO, study design, setting, time frame) and report characteristics (such as years considered, language, publication status) to be used as criteria for eligibility for the review	5,6,7
Information sources	#9	Describe all intended information sources (such as electronic databases, contact with study authors, trial registers or other grey literature sources) with planned dates of coverage	9,10
Search strategy	#10	Present draft of search strategy to be used for at least one electronic database, including planned limits, such that it could be repeated	9,14
Study records - data management	#11a	Describe the mechanism(s) that will be used to manage records and data throughout the review	10
Study records - selection process	#11b	State the process that will be used for selecting studies (such as two independent reviewers) through each phase of the review (that is, screening, eligibility and inclusion in meta-analysis)	10,11

For peer review only

Study records - data collection process	#11c	Describe planned method of extracting data from reports (such as piloting forms, done independently, in duplicate), any processes for obtaining and confirming data from investigators	10
Data items	#12	List and define all variables for which data will be sought (such as PICO items, funding sources), any pre-planned data assumptions and simplifications	10,11
Outcomes and prioritization	#13	List and define all outcomes for which data will be sought, including prioritization of main and additional outcomes, with rationale	8,9
Risk of bias in individual studies	#14	Describe anticipated methods for assessing risk of bias of individual studies, including whether this will be done at the outcome or study level, or both; state how this information will be used in data synthesis	11
Data synthesis	#15a	Describe criteria under which study data will be quantitatively synthesised	11,12
Data synthesis	#15b	If data are appropriate for quantitative synthesis, describe planned summary measures, methods of handling data and methods of combining data from studies, including any planned exploration of consistency (such as I2, Kendall's τ)	11
Data synthesis	#15c	Describe any proposed additional analyses (such as sensitivity or subgroup analyses, meta-regression)	11,12
Data synthesis	#15d	If quantitative synthesis is not appropriate, describe the type of summary planned	12
Meta-bias(es)	#16	Specify any planned assessment of meta-bias(es) (such as publication bias across studies, selective reporting within studies)	N/A
Confidence in cumulative evidence	#17	Describe how the strength of the body of evidence will be assessed (such as GRADE)	11,12

The PRISMA-P checklist is distributed under the terms of the Creative Commons Attribution License CC-BY 4.0. This checklist was completed on 08. February 2020 using https://www.goodreports.org/, a tool made by the EQUATOR Network in collaboration with Penelope.ai

For peer review only

Anticoagulation strategies in critical care for the treatment of atrial fibrillation: a protocol for a systematic review and meta-analysis

Journal:	*BMJ Open*
Manuscript ID	bmjopen-2020-037591.R2
Article Type:	Protocol
Date Submitted by the Author:	27-Sep-2020
Complete List of Authors:	Johnston, Brian; University of Liverpool Faculty of Health and Life Sciences,<br>Nelson, Alexandra; University of Liverpool Faculty of Health and Life Sciences<br>Waite, Alicia; University of Liverpool Institute of Ageing and Chronic Disease<br>Lemma, Gedeon; University of Liverpool Faculty of Health and Life Sciences<br>Welters, Ingeborg; University of Liverpool Institute of Ageing and Chronic Disease,
**Primary Subject Heading**:	Intensive care
Secondary Subject Heading:	Cardiovascular medicine, Anaesthesia, Pharmacology and therapeutics, Haematology (incl blood transfusion), Evidence based practice
Keywords:	INTENSIVE & CRITICAL CARE, Adult intensive & critical care < ANAESTHETICS, CARDIOLOGY, Anticoagulation < HAEMATOLOGY

SCHOLARONE™ Manuscripts

I, the Submitting Author has the right to grant and does grant on behalf of all authors of the Work (as defined in the below author licence), an exclusive licence and/or a non-exclusive licence for contributions from authors who are: i) UK Crown employees; ii) where BMJ has agreed a CC-BY licence shall apply, and/or iii) in accordance with the terms applicable for US Federal Government officers or employees acting as part of their official duties; on a worldwide, perpetual, irrevocable, royalty-free basis to BMJ Publishing Group Ltd ("BMJ") its licensees and where the relevant Journal is co-owned by BMJ to the co-owners of the Journal, to publish the Work in this journal and any other BMJ products and to exploit all rights, as set out in our licence.

The Submitting Author accepts and understands that any supply made under these terms is made by BMJ to the Submitting Author unless you are acting as an employee on behalf of your employer or a postgraduate student of an affiliated institution which is paying any applicable article publishing charge ("APC") for Open Access articles. Where the Submitting Author wishes to make the Work available on an Open Access basis (and intends to pay the relevant APC), the terms of reuse of such Open Access shall be governed by a Creative Commons licence – details of these licences and which Creative Commons licence will apply to this Work are set out in our licence referred to above.

Other than as permitted in any relevant BMJ Author's Self Archiving Policies, I confirm this Work has not been accepted for publication elsewhere, is not being considered for publication elsewhere and does not duplicate material already published. I confirm all authors consent to publication of this Work and authorise the granting of this licence.

For peer review only

Anticoagulation strategies in critical care for the treatment of atrial fibrillation: a protocol for a systematic review and meta-analysis

Authors

1) Brian W Johnston[1,2], 2) Alexandra Nelson[2], 3) Alicia C Waite[1,2], 4) Gedeon Lemma[2], 5) Ingeborg D Welters[1,2]

Corresponding author: Brian W Johnston, c/o Intensive Care Unit, Royal Liverpool and Broadgreen University Hospital, Prescott Street, L78XP, Contact Telephone: 0151 706 2410

Author Affiliations

1. University of Liverpool and The Royal Liverpool University Hospital, Liverpool Healthcare Partners, Liverpool, UK.
2. The University of Liverpool, Liverpool, UK.

Author contact details

1. Dr Brian. W Johnston, brian.johnston@liverpool.ac.uk, ORCID: 0000-0003-1634-3297
2. Miss Alexandra Nelson, hlanels2@liverpool.ac.uk
3. Dr Alicia C Waite, Alicia.Waite@liverpool.ac.uk, ORCID: 0000-0001-8734-994X
4. Mr Gedeon Lemma, hlglemma@liverpool.ac.uk
5. Prof Ingeborg D Welters, Ingeborg.welters@liverpool.ac.uk, ORCID: 0000-0002-3408-8798

Keywords

Atrial Fibrillation, Critical Illness, Intensive Care Unit, Anticoagulation,

Prospero Trials Registration Number: CRD42020158237

For peer review only

ABSTRACT

Introduction

Atrial fibrillation is the most common cardiac arrhythmia in critically ill patients and is associated with an increased risk of thromboembolic events and mortality. Oral anticoagulation for thromboembolism prophylaxis is a key component of managing atrial fibrillation in the general population and is recommended by NICE guidelines. However, assessment tools used to aid decision making about anticoagulation have not yet been validated in the critical care setting. There is a paucity of data assessing the impact of anticoagulation strategies on clinical outcomes in critically ill patients with atrial fibrillation. We present a protocol for a systematic review and meta-analysis to evaluate the effectiveness of anticoagulation strategies for atrial fibrillation used specifically in critical care.

Methods and analysis

We will conduct a systematic review of the literature by searching MEDLINE, EMBASE, CENTRAL and PubMed databases for articles published from January 1990 to October 2019. Studies reporting anticoagulation strategies for AF in adults (>18 years) admitted to a general critical care setting will be assessed for inclusion. Outcomes of interest will include, 1) percentage of patients commenced on anticoagulation in critical care for atrial fibrillation, 2) incidence of thromboembolism, 3) incidence of bleeding events, 4) ICU mortality, 5) hospital mortality, 6) ICU length of stay and 7) hospital length of stay. We will conduct a meta-analysis of trials. Risk of bias will be assessed using the Cochrane Risk of Bias tool for randomised trials or the Newcastle-Ottawa Risk of Bias assessment tool for non-randomised studies. This protocol and subsequent systematic review will be reported following the Preferred Reporting Items for Systematic Reviews and Meta-Analyses checklist.

Ethics and dissemination

This proposed systematic review will include data extracted from published studies, therefore ethical approval is not required. The results of this review will be published in clinical specialty journals and presented at international meetings and conferences.

Prospero Trial Registration Number: CRD42020158237

For peer review only

Strengths and limitations of this study

Strengths:

- This systematic review follows the Preferred Reporting Items for Systematic Review and Meta-Analysis Protocols guidelines.
- Study selection, data extraction and risk of bias assessment will be performed independently by two researchers, ensuring all relevant studies are included without personal biases.
- There are no language publication restrictions to this systematic review eliminating language bias.

Limitations:

- Non-randomised studies will be included in this review potentiating a high risk of bias in included studies.
- Given the particular risk for thromboembolic events in critically ill patients, we will only include studies on patients receiving anticoagulation during their critical care admission.

INTRODUCTION

Background

Atrial fibrillation (AF) is the most common cardiac arrhythmia in the critical care population. AF is characterised by erratic conduction of electrical impulses and uncoordinated contraction of the atria which ultimately increases the risk of heart failure, stroke and death [1,2]. The reported incidence of new-onset AF (NOAF) in critically unwell patients admitted to ICU has been estimated to be between 1.8% to 10% [1]. However, a more recent study reported NOAF in 418 of 1782 (23%) of patients admitted to ICU and was associated with increased hospital mortality [2][3]. AF can trigger rapid ventricular rates resulting in haemodynamic compromise and a loss of cardiac output. This may contribute to acute decompensation of already unstable critically ill patients and eventually lead to the increased morbidity and mortality associated with AF in critical care [2,4].

In addition to the traditional risk factors for the development of AF, including advanced age, hypertension, ischaemic heart disease, heart failure and valvular disease that predispose to pre-existing AF (PEAF); there are

For peer review only

factors related to being critically ill that predispose patients to the development of NOAF [1,2]. These factors include electrolyte abnormalities, hypoxaemia, adrenergic overstimulation, progressive autonomic dysfunction, acute systemic inflammation, sepsis and shock [1,2]. Changes in autonomic activity resulting from vasopressor administration as well as electrolyte disturbances are frequent among critically ill patients and can lead to increased atrial ectopic impulses and subsequent NOAF [4]. There is also evidence that central venous catheters induce mechanical irritation of the atria and may be a contributing factor in the development of NOAF [1]. The high incidence of NOAF in critical care is likely due to a combination of these traditional and critical illness specific related risk factors [4].

Both PEAF and NOAF are associated with a myriad of complications in critical care. As a result of inefficient atrial systole, the reduced blood flow velocity in the left atria predisposes patients with AF to cardiac and systemic emboli [5]. Embolic events, such as ischaemic stroke, are common and disabling complications of AF and cause a significant disease burden [6]. AF in the critical care setting is associated with a twofold increased risk of stroke and a two to fivefold increased risk of mortality [2,7].

Oral anticoagulation for thromboembolism prophylaxis is a key component of managing AF in the general population [8]. In the United Kingdom, clinicians are guided in the management of NOAF by the recommendations from the National Institute for Health and Care Excellence (NICE). With regards to anticoagulation for NOAF, NICE recommends the use of validated tools assessing thromboembolic risk (e.g. CHA_2DS_2-VASc) and bleeding risk (e.g. HASBLED) to stratify patients that may benefit from systemic anticoagulation through prevention of thromboembolic events such as stroke [8]. However, the risk-benefit tools used to aid decision making about anticoagulation, such as the CHA_2DS_2-VASc and HASBLED scores, have not been validated in critical care populations [9,10]. Decisions around anticoagulation strategies in critical care are complex and challenging. Critically unwell patients are at a significantly increased risk of bleeding, but may also of hypercoagulable, due to the abnormal haemostasis that is associated with critical illness [10]. Factors contributing to the acquired coagulopathy in critical care include severe sepsis, thrombocytopenia, haemodilution of clotting factors following blood transfusion, disseminated intravascular coagulation (DIC), acute kidney injury (AKI) and liver failure [11]. The potential need for urgent procedures or insertion of

For peer review only

invasive devices, such as arterial lines and central venous catheters, poses an additional challenge in effectively anticoagulating these patients [12]. Combined with the added effect of anticoagulation itself, the high risk of bleeding may preclude safe therapeutic anticoagulation for AF in critical care patients. Guidelines from NICE recommend anticoagulation with Heparin in patients presenting with NOAF that are receiving no or subtherapeutic anticoagulation. Furthermore, they recommend that patients who have failed to achieve stable sinus rhythm within 48 hours or have risk factors for the recurrence of NOAF should be offered oral anticoagulation long term. Despite these recommendations a nationwide survey of intensive care clinicians revealed that 63.8% of clinicians would not routinely anticoagulate critically ill patients with NOAF while 30.8% would consider anticoagulation if NOAF persisted beyond 72 hours rather than the recommended 48 hours by NICE [13]. Furthermore, 98% of critical care clinicians revealed that they would be happy to administer Heparin as anticoagulation but not an oral anticoagulation [13]. The variation in practice likely represents the unique challenges of managing NOAF in critically unwell patients, in which administration of oral anticoagulants may not possible in sedated patients or via nasogastric tube and highlights the need for guidelines specific to critically ill patients.

Why is it important to undertake this review?

There is a paucity of data assessing the impact of anticoagulation strategies on clinical outcomes in critically unwell patients with AF [14]. A consensus on an effective anticoagulation strategy for thromboembolism prophylaxis has not yet been reached and current recommendations regarding anticoagulation therapy are largely based on observational studies and expert opinion [4]. We therefore have designed a protocol for a systematic review and meta-analysis of the literature around the anticoagulation for AF in the critically unwell and critical care setting. We will assess the existing literature to define anticoagulation strategies in critical care for both NOAF and PEAF. We anticipate that the results of this review will highlight areas were evidence is lacking, trigger further research and contribute to the development of new guidelines specific to the management of anticoagulation in patients with AF in the critical care setting.

Objective

For peer review only - http://bmjopen.bmj.com/site/about/guidelines.xhtml

To conduct a systematic review and meta-analysis of the literature to determine the use and effectiveness of anticoagulation strategies for AF in critical care and identify the associated risks and benefits of therapeutic anticoagulation for AF.

METHODS AND ANALYSIS

This systematic review will be conducted in accordance with The Cochrane Collaboration principles of Systematic Reviews and will be reported following the Preferred Reporting Items for Systematic Reviews and Meta-Analyses (PRISMA) guidelines [15,16].

This protocol has been registered with the International Prospective Register of Systematic Reviews (PROSPERO) database. PROSPERO registration number: CRD42020158237

Inclusion criteria

Type of studies

All quantitative studies that report anticoagulation strategies for AF, in an adult critical care setting will be assessed for inclusion. Eligible studies will include randomised and non-randomised studies.

Eligible studies must include both a cohort of patients with AF who were anticoagulated and a cohort of patients with AF who were not anticoagulated. We will include studies in patients diagnosed with NOAF (including paroxysmal AF) or PEAF (including permanent AF) by rhythm classification by continuous ECG monitoring or 12 lead ECG.

We will include studies conducted in level 2 and level 3 critical care settings as defined by Marshall et al [17]. We will include studies enrolling patients from general medical, general surgical or mixed mixed/surgical patients.

Phenomenon of interest

For peer review only

163 Studies must include patients who have been in AF for >48 hours, based on the NICE guidelines on initiating anticoagulant treatment [8], and may include both PEAF and NOAF. Studies must describe outcomes associated with the chosen anticoagulation strategy including the outcomes of interest described below.

Population

Studies that include adult patients admitted to the ICU types specified above will be assessed for inclusion. For the purpose of this review, an adult is defined as ≥18 years.

Type of intervention and comparator(s)

Interventions:

Eligible studies will include any of the following treatments:

1) Warfarin
2) Any other vitamin K antagonist
3) Unfractionated heparin (UFH)
4) Treatment dose low molecular weight heparin (LMWH)
5) Factor Xa inhibitors (e.g Edoxaban, Apixaban, Rivaroxaban)
6) Direct thrombin (Factor IIa) inhibitors (Dabigatran, Inogatran, Melagatran, Argatroban)
7) Any combination of the above interventions

Comparators:

Comparators will include:

1) Any of the interventions above
2) Placebo
3) Standard care where it does not include anticoagulation

Exclusion criteria

For peer review only - http://bmjopen.bmj.com/site/about/guidelines.xhtml

Types of studies

Qualitative studies, case studies, editorials, letters, abstract only reports, reviews and commentaries that do not include original information will be excluded from this review.

Studies of cohorts that have undergone or plan to undergo cardiothoracic surgery, permanent pacemaker insertion or surgical ablation will be excluded. Studies based on service-specific ICUs, for example cardiac, cardiothoracic surgical or neurosurgical units, will be excluded in addition to studies based on acute medical units or in emergency departments.

Phenomenon of interest

Studies will be excluded if patients have been commenced on any anticoagulation therapy for a reason other than AF and cannot be disaggregated from the entire cohort. Studies that include patients with an inherited or pre-existing bleeding disorder or clotting disorder will also be excluded.

Population

Studies of participants under 18 years, pregnant women and patients with clear contraindication to anticoagulation therapy for example intracranial haemorrhage, overt bleeding or allergy to anticoagulant medication will be excluded.

Outcome measures

Primary outcome measure:

1) Percentage of patients commenced on anticoagulation for AF (NOAF and/or PEAF) in a critical care setting including but not limited to warfarin, LMWH, UFH, Factor Xa inhibitors, Factor IIa inhibitors.

Secondary outcome measures:

1) Incidence of thromboembolic events (defined as stroke, mesenteric ischaemia, acute limb ischaemia, pulmonary embolism) during critical care admission to identify the outcomes of anticoagulation therapy in the critical care setting.

For peer review only

2) Incidence of any in hospital thromboembolic events (defined as stroke, mesenteric ischaemia, acute limb ischaemia, pulmonary embolism).
3) Development of a major haemorrhage event (defined clinically as haemorrhage leading to death, signs of shock, requirement for blood transfusion or urgent endoscopic / surgical intervention) [18].
4) ICU length of stay (LOS)
5) ICU mortality
6) 28-day mortality
7) 90-day mortality
8) 365-day mortality
9) Use of risk stratification scores for anticoagulation decision making (such as CHA_2DS_2-VASc and HASBLED)
10) Severity scores of critical illness (e. g. APACHE II and SOFA)

Search strategy

We will engage the services of a health information specialist, and a comprehensive broad literature search will be conducted. Medical subject headings will be used to identify papers that matched the search index and relevant selected free text terms will also be used.

The search strategies identified will focus on the population (critically ill), the participants (patients with AF) and the intervention (any anticoagulation strategy mentioned previously). The search strategy will not be limited by outcomes studied in order to broaden the scope of eligible papers.

Database searches

We will search the following databases for studies published between January 1990 and October 2019;

1) MEDLINE Ovid, Medline Ovid Epub Ahead of Print, and Medline Ovid In-Process
2) EMBASE Ovid

For peer review only

3) The Cochrane Central Register of Controlled Trials

4) PUBMED

5) ClinicalTrials.gov

MEDLINE, EMBASE and PUBMED will be access via NICE Healthcare Database Advanced Search (HDAS) using OpenAthens. We will screen referenced papers following full text screening and any review articles identified during the screening process. We will screen for relevant conference proceedings and include any potentially relevant studies. A full description of the search strategy that will be used in HDAS (supplementary file).

Citation management and screening

Results from searches in all databases will be exported to Endnote X9 (Clarivate analytics) and duplicates will be removed. All citations will be imported into the Covidence systematic review platform (Veritas Health Innovation, Melbourne, Australia) [19]. Titles and abstracts will be screened in duplicate by two independent reviewers for relevance to the review and eliminated as appropriate. Full text articles that are not excluded after title and abstract screening, will be screened against the inclusion and exclusion criteria by two independent reviewers. Any discrepancies or conflicts in the screening process will be resolved by discussion and subsequent input of a third reviewer. Primary reason for exclusion will be documented in Covidence and the screening process will be documented in a PRISMA flowchart (Figure 1).

Data extraction and management

Data will be extracted in duplicate by two independent reviewers using a standardised, pre-piloted data extraction form.

Information, including the following characteristics, will be extracted from studies:

1) Study design and methodology, including title, authors, journal, publication date, study type, study period and number of participants

2) All inclusion criteria previously mentioned

For peer review only

3) Population characteristics, including age, sex, ICU admission diagnosis, location, co-morbidities, illness severity scores
4) Recruitment procedures
5) Interventions
6) The primary and secondary outcome measures and reported findings of the study

Any discrepancies within data extraction will be resolved through discussion and consultation with a third reviewer. We will contact authors if clarification regarding the data or methodology is required. If data cannot be obtained from the authors, the impact will be discussed as a limitation of the review.

Risk of bias assessment

Risk of bias will be assessed using a modified Newcastle-Ottawa Scale (mNOS), a scoring system for non-randomised trials [20]. mNOS uses a system by which the study is judged on three broad perspectives: the selection of the study groups, the comparability and the ascertainment of outcome of interest.

Included randomised controlled trials will be assessed with the revised Cochrane Collaborations Risk of Bias (RoB 2) tool using the criteria outlined in the Cochrane Handbook of Systematic Reviews of Interventions [21]. The RoB 2 tool assesses the following domains: selection, comparability, outcome performance and will be presented in table format.

Data Synthesis and Analysis

A description of included studies will be reported in evidence tables and discussed in the text. We will report participant characteristics, interventions, clinical outcomes and methodological quality.

Primary outcomes will be reported as the percentage of patients that received anticoagulation for any AF (NOAF and PEAF) in critical care. If sufficient data allows, we will report the percentage of patients commenced on anticoagulation according to type of AF (NOAF and/or PEAF).

For peer review only

We anticipate that the majority of studies included in our systematic review and meta-analysis will be case control studies. Therefore, we will present dichotomous data as number of participants experiencing the outcome with OR and 95% confidence intervals [22]. For continuous outcome data, we will extract arithmetic means and standard deviations with 95% confidence intervals for each outcome, together with the numbers analysed in each group. We will also extract medians and ranges where provided.

Data from individual studies will be pooled and presented visually by Forest plot. We anticipate studies to vary significantly, therefore we will undertake a meta-analysis of included studies by using a random effects model with 95% CI to estimate pooled estimates of effect. We will assess for heterogeneity between studies using the Cochran's Q test and report the degree of heterogeneity using the I^2 statistic in RevMan (Review Manager, 2014). Heterogeneity will be considered low if values are below 25%, moderated if values are between 25% and 75% and high if values are above 75%. Suitability for meta-analysis will be determined by the degree of clinical, statistical and methodological heterogeneity observed between studies. We will explore heterogeneity between studies by subgroup analysis assessing a number of factors such as study design, intervention and participants. Where there is a high degree of heterogeneity (>75%) that cannot be explained by clinical or methodological variation, we will not undertake a meta-analysis of results. If we are unable to pool studies due to heterogeneity, we will describe the findings via a narrative synthesis. Meta-analysis will be conducted using the Cochrane Collaborations RevMan (Review Manager, 2014) software.

We will assess the certainty of our evidence for each outcome according to the Grading of Recommendations Assessment, Development and Evaluation (GRADE) approach [23]. GRADE methodology assesses the certainty in evidence across the domains of risk of bias, consistency, directness, precision and publication bias. The certainty of evidence will be reported as high, moderate, low or very low.

Subgroup analysis

If we extract sufficient data, we will undertake subgroup analysis, assessing the following:

1. Type of anticoagulant therapy
2. NOAF versus PEAF
3. Sepsis versus non-sepsis

For peer review only

4. Illness severity scores (APACHE II score, SOFA score)
5. Risk scores for bleeding and thromboembolic events (HASBLED, CHA_2DS_2-VASc respectively)
6. Medical versus surgical ICU admission diagnosis

Patient and public involvement

This protocol will use previously published data. As such there will be no patient and public involvement in the design of the study, interpretation of the results. We will disseminate the results of this review to any interested patients and it will be freely accessible in open access journals.

Ethics and dissemination

This proposed systematic review will not include primary data and will extract data from published studies, therefore ethical approval is not required. The results of this review will be disseminated through publication in clinical specialty journals, including open access journals, and various medias including international meetings, conferences, congresses and symposiums.

Discussion

This systematic review will assess the evidence available for anticoagulation strategies used in the management of AF in critically ill patients. AF affects up to 25% of critical care admissions, hence the need for systematic evaluation of anticoagulation strategies used in critical care and their impact on patient outcomes [24]. AF is associated with an increased risk of thromboembolic complications, but scoring systems used to aid decision making on whether and how-to anticoagulate patients with AF have not been validated for use in the critical care setting. This review will enable a comparison of the anticoagulation strategies used for both PEAF and NOAF in critical care and aims to assess outcomes including thromboembolic events, haemorrhagic events, length of stay (LOS) in critical care and mortality on ICU. Critically ill patients have a particularly high risk of thromboembolic events, which could theoretically be ameliorated by administering anticoagulant therapy during critical illness. A limitation of our protocol is that results will not be generalizable to patients who are clinically stable after having been critically ill.

For peer review only - http://bmjopen.bmj.com/site/about/guidelines.xhtml

Based on a preliminary search of the literature, we believe that this is the first systematic review to consider anticoagulation strategies used in the treatment of AF in the critically ill patient cohort. We aim for this review to provide an evidence base upon which recommendations can be made, to ultimately reduce the impact of AF on patient outcomes during and after critical care admission. We will publish the results of this review in clinical specialty journals and we present it at international meetings and conferences. We will also make our report available via our web- based repository and update our PROSPERO entry with the web address details.

For peer review only

FOOTNOTES

Author’s contribution

The protocol was conceived and designed by IW, BWJ and AW. AN will conduct primary screening and data collection, reviewed by BWJ and GL. Data extraction, analysis and preparation of the manuscript will be conducted by AN and BWJ. IW, BWJ, AW, GL and AN will read and approve the final manuscript.

Funding

This research received no specific grant from any funding agency in the public, commercial or not-for-profit sectors.

Conflicts of interest

None declared.

Data Sharing Statement

All data is included in the manuscript.

Provenance and peer review

Not commissioned; externally peer reviewed.

Patient consent

Not required.

For peer review only - http://bmjopen.bmj.com/site/about/guidelines.xhtml

Figure Legend

Figure 1: PRISMA flowchart of studies selected in the systematic review

REFERENCES

1. Malik A, Candilio L, Hausenloy DJ. Atrial fibrillation in the intensive care setting. *Journal of the Intensive Care Society*. 2013;14(2):141-9.

2. Bosch NA, Cimini J, Walkey AJ. Atrial Fibrillation in the ICU. *Chest*. 2018;154(6):1424-34.

3. Klouwenberg K, Frenchken JF, Kuipers S, Ong DS, Peelen LM, Vught LA, Schultz MJ, Poll T, Bonten MJ, Cremer OL, and MARS Consortium. Incidence, Predictors, and Outcomes of New-Onset Atrial Fibrillation in Critically Ill Patients with Sepsis. A Cohort Study. Am J Respir Crit Care Med. 2017;195(2):205-211

4. Walkey AJ, Hogarth DK, Lip GYH. Optimizing Atrial fibrillation management from ICU and beyond. *Chest*. 2015;148(4):859-64.

5. Nadzeya Kuzniatsova, Lip GYH. Prevention of Thromboembolism in Atrial Fibrillation Patients. *European Cardiology Review*. 2011;7(1):37-43.

6. Gorczyca I, Wozakowska-Kaplon B, Starzyk K et al. Evaluation of the recommended prevention of thrombosis in hospitalised patients with atrial fibrillation and high thromboembolism risk. *Kardiol Pol.* 2018;76(3):625-32.

7. Arrigo M, Bettex D, Rudiger A. Management of atrial fibrillation in critically ill patients. *Crit Care Res Pract*. 2014;2014:840615-.

8. Gilligan DM, Ellenbogen K, Epstein E. Atrial Fibrillation: Management 2014 [updated August 2014.] Available from: https://www.nice.org.uk/guidance/cg180

9. Kanji S, Williamson DR, Yaghchi BM et al. Epidemiology and management of atrial fibrillation in medical and noncardiac surgical adult intensive care unit patients. *J Crit Care.* 2012;27(3):326.e1-8.

10. Poli D, Antonucci E, Pengo V et al. Comparison of HAS-BLED and HAS-BED Versus $CHADS_2$ and CHA_2DS_2VASC Stroke and Bleeding Scores in Patients With Atrial Fibrillation. *American Journal of Cardiology*. 2017;119(7):1012-6.

11. Retter A, Barrett NA. The management of abnormal haemostasis in the ICU. *Anaesthesia*. 2015;70 Suppl 1:121-7, e40-1.

For peer review only

12. Sibley S, Muscedere J. New-onset atrial fibrillation in critically ill patients. *Can Respir J.*2015;22(3):179-82.

13. Chean CS, McAuley D, Gordon A, Welters ID. Current practice in the management of new-onset atrial fibrillation in critically ill patients: a UK-wide survey. PeerJ. 2017;5:e3716.

14. Darwish OS, Strube S, Nguyen HM et al. Challenges of anticoagulation for atrial fibrillation in patients with severe sepsis. *Ann Pharmacother*. 2013;47(10):1266-71.

15. Moher D, Liberati A, Tetzlaff J et al. Preferred reporting items for systematic reviews and meta-analyses: the PRISMA statement. *PLoS Med*. 2009;6(7):e1000097.

16. Hutton B, Salanti G, Caldwell DM et al. The PRISMA extension statement for reporting of systematic reviews incorporating network meta-analyses of health care interventions: checklist and explanations. *Ann Intern Med.* 2015;162(11):777-84.

17. Marshall JC, Bosco L, Adhikari NK, Connolly B, Diaz JV, Dorman T, et al. What is an intensive care unit? A report of the task force of the World Federation of Societies of Intensive and Critical Care Medicine. J Crit Care. 2017;37:270-6.

18. Roberts I, Shakur-Still H, Afolabi A, Akere A, Arribas M, Brenner A, et al. Effects of a high-dose 24-h infusion of tranexamic acid on death and thromboembolic events in patients with acute gastrointestinal bleeding (HALT-IT): an international randomised, double-blind, placebo-controlled trial. The Lancet. 2020;395(10241):1927-36.

19. Covidence systematic review software, Veritas Health Innovation, Melbourne, Australia. Available at www.covidence.org

20. Stang A. Critical evaluation of the Newcastle-Ottawa scale for the assessment of the quality of nonrandomized studies in meta-analyses. *Eur J Epidemiol*. 2010;25(9):603-5.

21. Higgins JPT, Thomas J, Chandler J et al. *Cochrane handbook for systematic reviews of interventions.* 2nd edition. Chichester (UK): John Wiley & Sons, 2019.

22. Higgins JPT, Li T, Deeks JJ. Chapter 6: Choosing effect measures and computing estimates of effect. In: Higgins JPT, Thomas J, Chandler J, Cumpston M, Li T, Page MJ, Welch VA (editors). *Cochrane Handbook for Systematic Reviews of Interventions* version 6.0 (updated July 2019). Cochrane, 2019. Available from www.training.cochrane.org/handbook.

For peer review only

426 23. Balshem H HM, Schunemann HJ, Oxman AD et al. GRADE guidelines: 3. Rating the quality of evidence. *J Clin Epidemiol*. 2011;64:401-6.

428 24. Shaver CM, Chen W, Janz DR et al. Atrial Fibrillation Is an Independent Predictor of Mortality in Critically Ill Patients. *Crit Care Med*. 2015;43(10):2104-11.

For peer review only

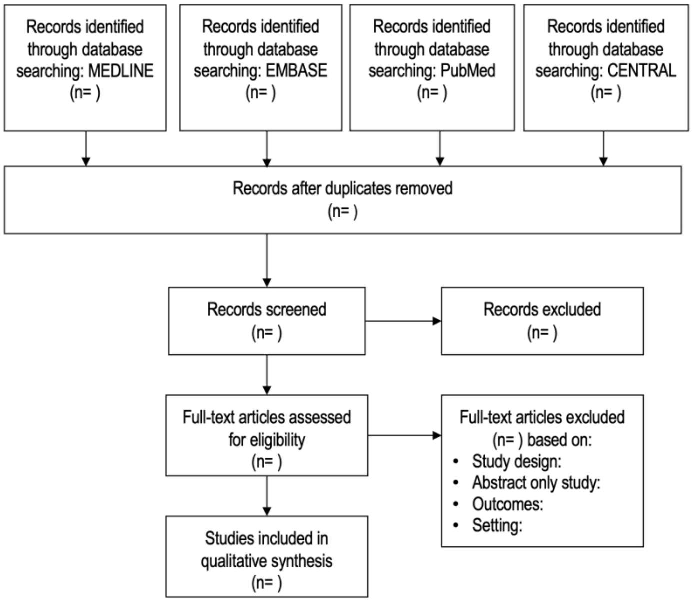

Figure 1 PRISMA flowchart of studies selected in the systematic review.

Figure 1. PRISMA flowchart of studies selected in the systematic review.

209x297mm (300 x 300 DPI)

Supplementary File: Search Strategy used to identify papers included in this review using the Healthcase Databases Advanced Search engine.

DATABASE	MESH TERMS	KEYWORDS (TI AND AB)
MEDLINE	ANTICOAGULANTS/ OR "4HYDROXYCOUMARINS"/ OR ABCIXIMAB/ OR ACENOCOUMAROL/ OR ANCROD/ OR ANTITHROMBINS/ OR BECAPLERMIN/ OR "BETA 2-GLYCOPROTEIN I"/ OR "BLOOD COAGULATION FACTOR INHIBITORS"/ OR "CITRIC ACID"/ OR DALTEPARIN/ OR "DERMATAN SULFATE"/ OR DEXTRANS/ OR DICUMAROL/ OR "EDETIC ACID"/ OR ENOXAPARIN/ OR "ETHYL BISCOUMACETATE"/ OR "FIBRIN FIBRINOGEN DEGRADATION PRODUCTS"/ OR GABEXATE/ OR HEPARIN/ OR "HEPARIN, LOWMOLECULAR-WEIGHT"/ OR HEPARINOIDS/ OR NADROPARIN/ OR "PENTOSAN SULFURIC POLYESTER"/ OR PHENINDIONE/ OR PHENPROCOUMON/ OR "PROTEIN C"/ OR "PROTEIN S"/ OR "SODIUM CITRATE"/ OR TINZAPARIN/ OR WARFARIN/ exp "BLOOD COAGULATION"/	(anticoagul* OR anti-coagul* OR warfarin OR heparin* OR antiplatelet* OR anti-platelet* OR "Direct Oral Anticoagul*" OR "DOAC*" OR "new oral anticoagul*" OR "NOAC*" OR apixaban OR dabigatran OR "dabigatran etexilate" OR rivaroxaban OR "vitamin K antagonist*" OR "VKA").ti,ab
	exp "ATRIAL FIBRILLATION"/	("atrial fibrillation" OR "AF" OR "atrial flutter*" OR "supraventricular tachy*" OR "SVT").ti,ab
	exp "CRITICAL CARE"/	("critical care" OR "Intensive care" OR "ITU" OR "critical* ill*" OR "high depend*" OR "intensive therapy").ti,ab
EMBASE	exp "ANTICOAGULANT AGENT"/	(anticoagul* OR anti-coagul* OR warfarin OR heparin* OR antiplatelet* OR anti-platelet* OR "Direct Oral Anticoagul*" OR "DOAC*" OR "new oral anticoagul*" OR "NOAC*" OR apixaban OR dabigatran OR "dabigatran etexilate" OR rivaroxaban OR "vitamin K antagonist*" OR "VKA").ti,ab
	exp "ATRIAL FIBRILLATION"/	("atrial fibrillation" OR "AF" OR "atrial flutter*" OR "supraventricular tachy*" OR "SVT").ti,ab
	"INTENSIVE CARE"/	("critical care" OR "Intensive care" OR "ITU" OR "critical* ill*" OR "high depend*" OR "intensive therapy").ti,ab
PUBMED		(anticoagul* OR anti-coagul* OR warfarin OR heparin* OR antiplatelet* OR anti-platelet* OR "Direct Oral Anticoagul*" OR "DOAC*" OR "new oral anticoagul*" OR "NOAC*" OR apixaban OR dabigatran OR "dabigatran etexilate" OR rivaroxaban OR "vitamin K antagonist*" OR "VKA").ti,ab
		("atrial fibrillation" OR "AF" OR "atrial flutter*" OR "supraventricular tachy*" OR "SVT").ti,ab
		("critical care" OR "Intensive care" OR "ITU" OR "critical* ill*" OR "high depend*" OR "intensive therapy").ti,ab

For peer review only

COCHRANE		(anticoag*)
		"atrial fibrillation" OR AF
		"Critical care" OR "Intensive care" OR "ITU" OR "high depend*" OR "HDU"

For peer review only

Reporting checklist for protocol of a systematic review.

Based on the PRISMA-P guidelines.

Instructions to authors

Complete this checklist by entering the page numbers from your manuscript where readers will find each of the items listed below.

Your article may not currently address all the items on the checklist. Please modify your text to include the missing information. If you are certain that an item does not apply, please write "n/a" and provide a short explanation.

Upload your completed checklist as an extra file when you submit to a journal.

In your methods section, say that you used the PRISMA-Preporting guidelines, and cite them as:

Moher D, Shamseer L, Clarke M, Ghersi D, Liberati A, Petticrew M, Shekelle P, Stewart LA. Preferred Reporting Items for Systematic Review and Meta-Analysis Protocols (PRISMA-P) 2015 statement. Syst Rev. 2015;4(1):1.

		Reporting Item	Page Number
**Title**			
Identification	#1a	Identify the report as a protocol of a systematic review	1
Update	#1b	If the protocol is for an update of a previous systematic review, identify as such	N/A
**Registration**			
	#2	If registered, provide the name of the registry (such as PROSPERO) and registration number	1
**Authors**			
Contact	#3a	Provide name, institutional affiliation, e-mail address of all protocol authors; provide physical mailing address of corresponding author	1
Contribution	#3b	Describe contributions of protocol authors and identify the guarantor of the review	14

For peer review only

**Amendments**			
	#4	If the protocol represents an amendment of a previously completed or published protocol, identify as such and list changes; otherwise, state plan for documenting important protocol amendments	N/A
**Support**			
Sources	#5a	Indicate sources of financial or other support for the review	14
Sponsor	#5b	Provide name for the review funder and / or sponsor	N/A
Role of sponsor or funder	#5c	Describe roles of funder(s), sponsor(s), and / or institution(s), if any, in developing the protocol	N/A
**Introduction**			
Rationale	#6	Describe the rationale for the review in the context of what is already known	3,4,5
Objectives	#7	Provide an explicit statement of the question(s) the review will address with reference to participants, interventions, comparators, and outcomes (PICO)	5
**Methods**			
Eligibility criteria	#8	Specify the study characteristics (such as PICO, study design, setting, time frame) and report characteristics (such as years considered, language, publication status) to be used as criteria for eligibility for the review	5,6,7
Information sources	#9	Describe all intended information sources (such as electronic databases, contact with study authors, trial registers or other grey literature sources) with planned dates of coverage	9,10
Search strategy	#10	Present draft of search strategy to be used for at least one electronic database, including planned limits, such that it could be repeated	9,14
Study records - data management	#11a	Describe the mechanism(s) that will be used to manage records and data throughout the review	10
Study records - selection process	#11b	State the process that will be used for selecting studies (such as two independent reviewers) through each phase of the review (that is, screening, eligibility and inclusion in meta-analysis)	10,11

For peer review only

Study records - data collection process	#11c	Describe planned method of extracting data from reports (such as piloting forms, done independently, in duplicate), any processes for obtaining and confirming data from investigators	10
Data items	#12	List and define all variables for which data will be sought (such as PICO items, funding sources), any pre-planned data assumptions and simplifications	10,11
Outcomes and prioritization	#13	List and define all outcomes for which data will be sought, including prioritization of main and additional outcomes, with rationale	8,9
Risk of bias in individual studies	#14	Describe anticipated methods for assessing risk of bias of individual studies, including whether this will be done at the outcome or study level, or both; state how this information will be used in data synthesis	11
Data synthesis	#15a	Describe criteria under which study data will be quantitatively synthesised	11,12
Data synthesis	#15b	If data are appropriate for quantitative synthesis, describe planned summary measures, methods of handling data and methods of combining data from studies, including any planned exploration of consistency (such as I2, Kendall's τ)	11
Data synthesis	#15c	Describe any proposed additional analyses (such as sensitivity or subgroup analyses, meta-regression)	11,12
Data synthesis	#15d	If quantitative synthesis is not appropriate, describe the type of summary planned	12
Meta-bias(es)	#16	Specify any planned assessment of meta-bias(es) (such as publication bias across studies, selective reporting within studies)	N/A
Confidence in cumulative evidence	#17	Describe how the strength of the body of evidence will be assessed (such as GRADE)	11,12

The PRISMA-P checklist is distributed under the terms of the Creative Commons Attribution License CC-BY 4.0. This checklist was completed on 08. February 2020 using https://www.goodreports.org/, a tool made by the EQUATOR Network in collaboration with Penelope.ai

For peer review only
